# The value of Protein Phosphatase Methylesterase 1 in diagnosis, prognosis and immunoregulation: from pan-cancer analysis to breast cancer verification

**DOI:** 10.3389/fimmu.2026.1770711

**Published:** 2026-03-10

**Authors:** Yiyang Wang, Yue Zhang, Yongxiang Li, Haotian Ma, Jiawei Zhao, Dilimulati Ismtula, Chenming Guo

**Affiliations:** 1 Department of Breast Surgery, Center of Digestive and Vascular, The First Affiliated Hospital of Xinjiang Medical University, Urumqi, China; 2 Thyroid and Breast Surgery Department of the People’s Hospital of Bayingolin Mongol Autonomous Prefecture, Korla, China

**Keywords:** breast cancer, pan-cancer, PPME1, prognosis, tumor immunity

## Abstract

**Background:**

The Protein Phosphatase Methylesterase 1 (PPME1) is a methylesterase specific to phosphatase 2A, a tumor suppressor, and plays a key role in tumor development. Its impact on pan-cancer diagnosis, prognosis, and immune regulation is still uncertain.

**Methods:**

We analyzed PPME1 expression across multiple carcinomas using TCGA, GEO, and other datasets, focusing on its transcriptional profile, prognostic value, genetic and epigenetic alterations, and immunological role. To substantiate our findings, we evaluated PPME1 expression and protein levels in breast cancer tissues through RT-qPCR, Western blotting, and immunohistochemical techniques. The role of PPME1 in tumor progression was evaluated *in vitro* using CCK-8, colony formation, wound healing, and Matrigel transwell assays, and *in vivo* using xenograft tumor models.

**Results:**

Our pan-cancer analysis demonstrates that PPME1 is upregulated in the majority of tumors and exhibits heterogeneous expression across immune and molecular subtypes. Elevated levels of PPME1 constitute an independent risk factor in various cancers, including BLCA, BRCA, HNSC, KICH, LIHC, and UVM, and are associated with a poor prognosis. It has moderate to high diagnostic accuracy and can impact the tumor microenvironment by modifying genomic stability, affecting immunotherapy outcomes, and altering the immune response from anti-tumor to tumor-promoting. Functional enrichment analysis indicates PPME1’s involvement in DNA damage and repair, pathway activation, substance metabolism, protein modification, and immune regulation. In breast cancer, PPME1 mRNA and protein levels are elevated in tumor tissues compared to adjacent normal tissue. Functional experiments revealed that suppression of PPME1 significantly inhibits the migration, invasion, and tumor growth of breast cancer cells, suggesting that PPME1 may serve as a potential therapeutic target for breast cancer progression.

**Conclusions:**

Overall, our data indicate that PPME1 acts as an oncogenic driver in multiple cancer types, promoting BRCA progression by enhancing pro-tumorigenic pathways.

## Introduction

Cancer cases are increasing quickly, and the high death rate significantly impacts human life (1, 2). Although diverse antitumor treatments—including surgery, radiofrequency ablation, chemotherapy, immunotherapy, and targeted therapies—have advanced, a definitive cure remains elusive (3). Consequently, comprehensive pan-cancer studies focusing on specific genes and their roles across malignancies are urgently needed. Such research endeavors are essential for yielding valuable insights and fostering the development of innovative strategies for cancer treatment.

Protein Phosphatase 2A (PP2A) is an essential tumor suppressor, with its modifications linked to various human cancers (4). PP2A regulates key signaling pathways like WNT, mTOR, and MAP kinases by dephosphorylating over 300 substrates, including c-MYC, p53, and β-catenin, thus affecting their activity and stability (5). The Protein Phosphatase Methylesterase 1 (PPME1) gene, located at the 11q13.4 locus on the chromosome, encodes a PP2A-specific methylesterase consisting of 386 amino acids, which facilitates the demethylation and subsequent inactivation of PP2A (6). PPME1 plays a crucial role in the development of multiple tumors, such as pancreatic (7, 8), thyroid (9), and prostate cancers (10). Despite this, research has predominantly concentrated on the regulatory role of PP2A, often neglecting the potential contributory role of PPME1 as a primary oncogenic factor. Consequently, a comprehensive pan-cancer analysis of PPME1 is imperative to elucidate its role and significance in cancer.

This study investigated PPME1’s expression and prognostic value across various cancers, its link to genomic heterogeneity, and its relationship with infiltrating lymphocytes. *In vivo* and *in vitro* experiments confirmed PPME1’s crucial role in breast cancer. These results provide a new basis and potential target for cancer diagnosis, treatment, and prognosis.

## Material and methods

### Data on PPME1 expression were collected and analyzed across a range of cancers

Data for 18,102 samples, comprising 33 cancer-related RNA sequencing profiles, were acquired and analyzed using the UCSC XENA platform (https://xenabrowser.net/datapages/). The RNA-seq data had been uniformly processed with the STAR pipeline and were provided in Transcripts Per Million (TPM) format. For differential expression analysis in subsequent studies, these data were transformed via log2(value + 1). Statistical analysis was performed using the “stats” and “car” R packages, and data were visualized with “ggplot2”.

Furthermore, the differential expression of PPME1 at the protein level was confirmed by analyses using the UALCAN (11) and HPA (12) databases.

We used the TISIDB (13) databases (http://cis.hku.hk/TISIDB/index.php) to analyze the links between PPME1 expression, molecular subtypes, and pan-cancer immunological subtypes.

### Investigating how PPME1 expression levels correlate with prognosis and diagnostic potential in pan-cancer

Using TCGA survival data, we explored how PPME1 expression correlates with cancer prognosis. We used Cox regression and Kaplan-Meier curves via the ‘survival’ and ‘survminer’ R packages, and visualized results with forest plots and Venn diagrams using ‘ggplot2’.

To evaluate PPME1’s diagnostic ability to distinguish tumor from normal tissues, we conducted ROC analysis with the ‘pROC’ package. An AUC near 1 indicates high diagnostic accuracy, with 0.7-0.9 as moderate and above 0.9 as strong diagnostic potential.

### Construct and calibrate nomograms

The evaluation of factors affecting patient outcomes was conducted through univariate and multivariate Cox regression analyses. Variables demonstrating a p-value below 0.05 in the univariate analysis were subsequently entered into the multivariate model. PPME1 expression was categorized at the median and treated as an independent variable. A nomogram was created from multivariate analysis parameters, and its predictive accuracy was evaluated using the C-index across 1,000 replicates. A calibration curve was subsequently drawn to evaluate the agreement between the forecasted results and the real survival outcomes.

### Examination of genomic variability of PPME1

Using the cBioPortal (14) database (https://www.cbioportal.org), we explored genetic changes in PPME1. We assessed alteration frequency, mutation types, and CNVs. And we analyzed the protein’s 3D structure for comprehensive genetic understanding.

The GSCA (15) database (http://bioinfo.life.hust.edu.cn/GSCA/#/) served as a tool to investigate CNV percentages in a range of cancer types. Additionally, we investigated the prognostic implications of CNV in PPME1 for cancer patients.

The study explored the association between PPME1 and essential genomic variability metrics, with data sourced from the Sangerbox 3.0 (16) online database (http://vip.sangerbox.com/).

### Analysis of the relationship between PPME1 expression and immunity

The ESTIMATE (17) algorithm was used to calculate StromalScore, ImmuneScore, and ESTIMATEScore. The ssGSEA algorithm was subsequently employed to evaluate the infiltration levels of 24 types of immune cells. Through the Timer2.0 (18) database, we assessed the connection between PPME1 expression and the infiltration of immune cells in tumors. Within the ‘Immune’ module, CD8+ T cells, CD4+ T cells, B cells, cancer-associated fibroblasts (CAFs), myeloid dendritic cells, natural killer (NK) cells, monocytes, macrophages, and myeloid-derived suppressor cells (MDSCs) were quantified using the EPIC, TIMER, XCELL, QUANTISEQ, CIBERSORT, and MCPCOUNTER algorithms. We obtained single-cell data in.h5 format and annotations from the TISCH (19) database, then processed and analyzed this data using the R packages “MAESTRO” and “Seurat”. We used t-SNE for cell clustering to visualize PPME1 expression across different cells.

### The biological significance of PPME1

To explore proteins that interact with PPME1, we employed the STRING (20) database (https://string-db.org/) to build a PPI network with proteins confirmed through experiments to bind with PPME1. We used the ‘Similar Genes Detection’ feature of the GEPIA2 (21) database (http://gepia2.cancer-pku.cn/#index) to verify the genes related to PPME1 and their correlations. A Venn diagram was then constructed to identify genes common to both PPME1-binding proteins and the related genes. To wrap up, we employed the ‘clusterProfiler’ and ‘org.Hs.eg.db’ packages for a functional enrichment analysis of PPME1. The relationship between PPME1 and cancer cell states was explored through the cancerSEA (22) database (http://biocc.hrbmu.edu.cn/CancerSEA/).

The ‘DESeq2’ package was utilized for differential gene expression analysis. All genes with associated “log2FoldChange” values were subsequently subjected to GSEA to investigate functional distinctions between the groups across multiple cancer cohorts. We utilized the gene set from the MSigDB (23) database (available at https://www.gsea-msigdb.org/gsea/index.jsp) and conducted the analysis 10,000 iterations. The results are depicted in a ridge diagram, illustrating the top 10 “Reactome pathways”.

### Patient tissue sample collection

This study involved collecting tumor and adjacent normal tissues from 16 BRCA patients who had surgery at Xinjiang Medical University from January to December 2025, adhering to specific criteria (no preoperative treatments). The ethics committee approved the informed consent (No. 230714-07). Post-surgery, tissues were split: one part was frozen for qRT-PCR and WB analysis, and the other was used for fixed sections. Data were obtained from triplicate experiments.

### Plasmid construction and transfection

Gene vectors for PPME1 knockdown were constructed by inserting three shRNA sequences (refer to **Supplementary Table 1**) into the pLKO.1-puro lentiviral vector, with successful cloning verified through sequencing.For PPME1 overexpression, the gene was inserted into the pCDH vector with a promoter, also verified by sequencing.

For transfection, 2μg of shRNA or overexpression plasmids were mixed with Lipofectamine 3000 (Invitrogen, Shanghai, China) and introduced into 80% confluent HEK-293T cells. After a 48-hour culture for viral particle production, the particles underwent collection, filtration, and concentration through ultracentrifugation. The supernatant was then extracted, and the particles were reintroduced into the full growth medium.

### Lentivirus infection and grouping

The MCF-7 and MDA-MB-231 cell lines were cultured to 80% confluence before being infected with a concentrated lentivirus. The medium was refreshed after 24 hours, and the cells were cultured for an additional 48 hours. The cells were categorized into four distinct groups: NC-KD (PPME1 knockdown with an empty vector), KD (PPME1 knockdown), NC-OE (PPME1 overexpression with a vector), and OE (PPME1 overexpression). Seventy-two hours post-infection, gene expression was assessed utilizing fluorescence microscopy. Prior to collection for subsequent experimental procedures, the cells were maintained under optimal conditions.

### RNA and protein extraction, real-time PCR and Western blot analysis

RNA was extracted using Trizol (Invitrogen, Shanghai) and detected with a SYBR Green PCR kit (Takara, Kyoto). Target genes (see **Supplementary Table 1**) were amplified in a 20 µL reaction with specific primers. The 2–ΔΔCT method was used for data analysis after conducting quantitative real-time PCR on an Applied Biosystems 7500 system.

Protein extraction from tissues and cells was performed using RIPA lysis buffer (Beyotime, Shanghai, China) supplemented with 1 mM phenylmethylsulfonyl fluoride (PMSF, Beyotime, Shanghai, China). The BCA assay kit (Beyotime, Shanghai, China) was utilized to quantify protein concentrations. Equal amounts of proteins (30μg per lane) were separated by 10% sodium dodecyl sulfate-polyacrylamide gel electrophoresis (SDS-PAGE) and transferred onto a 0.45-μm pore-size PVDF membrane (Millipore, Billerica, MA, USA, Catalog No. IPVH00010). The membrane was blocked with 5% non-fat milk in TBST buffer (20 mM Tris-HCl, 150 mM NaCl, 0.1% Tween-20) for 1 h at room temperature, then incubated overnight at 4 °C with a primary antibody targeting PPME1 (Abcam, USA, Catalog No. ab154569, dilution ratio 1:1000). An antibody against α-tubulin (Abcam, USA, Catalog No. ab7291, dilution ratio 1:5000) was employed as a loading control. After three washes with TBST, the membrane was incubated with horseradish peroxidase (HRP)-conjugated goat anti-rabbit IgG secondary antibody (Beyotime, Shanghai, China, Catalog No. A0208, dilution ratio 1:5000) for 1 h at room temperature. Protein bands were detected using ECL Plus Western Blotting Detection Reagents (Thermo Fisher Scientific, Waltham, MA, USA, Catalog No. 32106) and visualized on a Bio-Rad ChemiDoc XRS+ imaging system (Bio-Rad, Hercules, CA, USA).

### Immunohistochemical staining

The present study analyzed 16 pairs of paraffin-embedded breast cancer tissues and corresponding adjacent non-tumor tissues. Tissue sections (4 μm thick) were deparaffinized in xylene and rehydrated through a graded ethanol series (100%, 95%, 85%, 75%, 50%). Antigen retrieval was performed by boiling the sections in citrate buffer (pH 6.0, Beyotime, Shanghai, China) for 15 min in a microwave oven. Endogenous peroxidase activity was blocked with 3% hydrogen peroxide (H_2_O_2_) for 10 min at room temperature. Non-specific binding was blocked with 5% goat serum (Beyotime, Shanghai, China) for 30 min at room temperature. The sections were then incubated overnight at 4 °C with a specific anti-PPME1 antibody (Abcam, USA, Catalog No. ab154569, dilution ratio 1:200). After three washes with PBS, the sections were incubated with HRP-conjugated goat anti-rabbit IgG secondary antibody (Beyotime, Shanghai, China, Catalog No. A0208, dilution ratio 1:200) for 30 min at room temperature. The immunoreactive signals were developed using 3,3’-diaminobenzidine (DAB) chromogen solution (Beyotime, Shanghai, China, Catalog No.P0202) and counterstained with hematoxylin for 2 min. The sections were then dehydrated, cleared, and mounted with neutral balsam. Two blinded pathologists independently evaluated the staining. The histoscore, calculated by multiplying intensity (0–3) and proportion (1-4) scores, ranged from 0 to 12. Scores of 0–5 indicated low expression, while 6–12 indicated high expression.

### CCK-8 cell proliferation assay

Cell proliferation was assessed using a CCK-8 assay (Dojindo, Japan). After seeding cells and culturing them for five days, CCK-8 reagent was added, and after a 1.5-hour incubation, absorbance was measured at 450 nm with a Tecan microplate reader from Switzerland. The results are presented as a line graph illustrating the proliferation trends.

### Colony formation assay

Cancer cells were treated to create a single-cell suspension, counted, and 500 cells were placed in each well of a 6-well plate. Following 1–2 weeks of culture, they were fixed and stained. After rinsing and air-drying, colonies were photographed and counted.

### Transwell migration and invasion assay

Transwell chambers were used for migration assays, with Matrigel coating for invasion assays only. Cells were trypsinized and resuspended in serum-free medium at a density of 5 × 10^4^ cells/mL. A total of 200 μL of the cell suspension was seeded into the upper chamber of the Transwell insert (8-μm pore size). The lower chamber was filled with 600 μL of complete medium supplemented with 10% fetal bovine serum to serve as the chemoattractant. After a 24-hour incubation at 37 °C with 5% CO_2_, non-migrated or non-invaded cells were eliminated. Cells on the lower surface were fixed, stained, and then imaged and counted under a microscope at 200× magnification.

### Wound healing assay

In 6-well plates, cells were grown until fully confluent, and a scratch was made with a sterile pipette tip. The cells were then washed and cultured in a medium without serum. Images of the wound were taken using an inverted microscope. The wound area was quantified using ImageJ software, and the cell migration rate was determined by the formula: [(Initial wound area - Wound area at a specific time point)/Initial wound area] × 100%.

### Animal studies

Animal experiments were approved by the Ethics Committee of the First Affiliated Hospital of Xinjiang Medical University (A240301-194). All animal experimental methods were performed in accordance with the relevant guidelines and regulations for the care and use of laboratory animals. The study design, animal handling, experimental procedures, and euthanasia methods strictly adhered to the institutional ethical standards and national regulatory requirements for animal research.

Eighteen 6-week-old NCG mice were obtained from Jiangsu Jichi Pharmaceutical Kang Biotechnology Co., LTD, with quarantine certificate B202411140401. The mice were divided into three groups of six. Cells were digested, counted, and resuspended in PBS, then mixed with Matrigel to a concentration of 5 × 10^6^ cells/100 μL and injected into the right armpit of the mice. Tumor dimensions were measured every three days starting from day 7 post-inoculation, and volume calculated using the formula: 0.5 × L × W². When the control group’s average tumor volume reached 1000 mm³ or humane endpoints were met, all mice were sacrificed for tumor analysis.

### Statistical analysis

Statistical analyses were conducted in R (version 4.2.1, https://www.r-project.org/), with data visualized using the “ggplot2” package. To evaluate differences between unpaired groups, the Mann-Whitney U test was employed, while paired samples were analyzed using the Wilcoxon signed-rank test. The associations between PPME1 expression and tumour heterogeneity or immune scores were analyzed via Spearman’s correlation. Statistical significance was established at a threshold of P < 0.05. Hazard ratios (HR) were computed along with 95% confidence intervals. To address the potential for false positives arising from multiple comparisons, the Benjamini-Hochberg procedure was employed to adjust p-values in the differential gene expression analysis, with the significance threshold determined at q < 0.05, corresponding to a false discovery rate (FDR) of less than 5%.

## Results

### Differential expression analysis of PPME1 across pan-cancer

**Figure 1:**
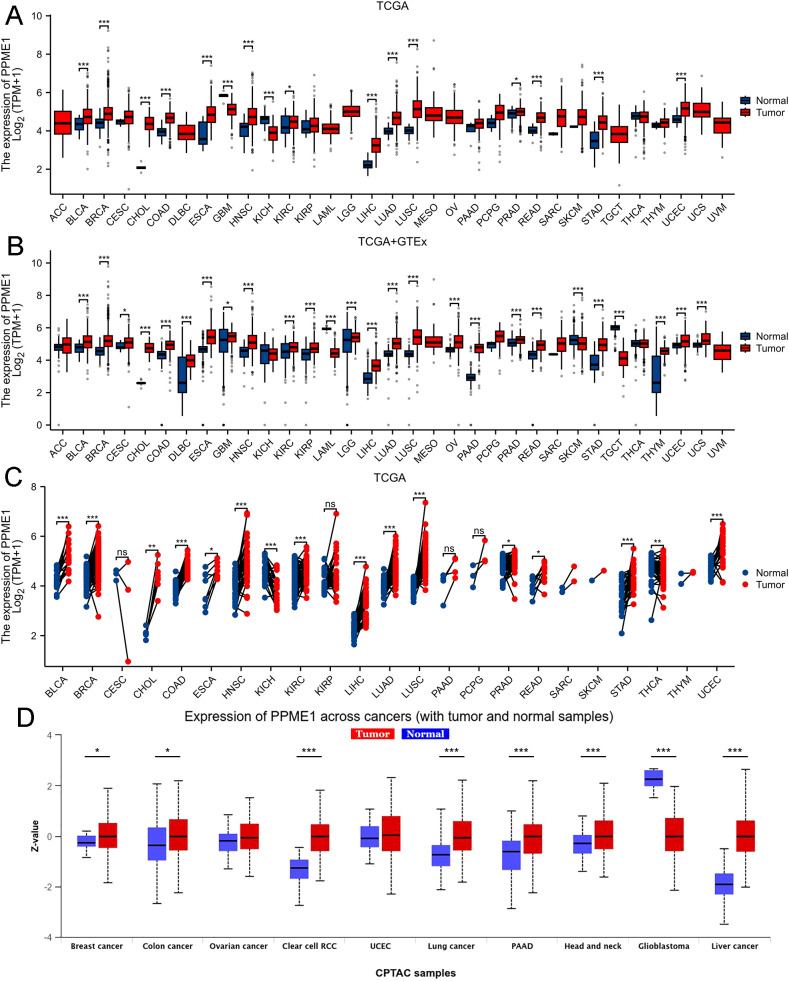
PPME1 expression variations across 33 cancer types. **(A)** Comparison of PPME1 mRNA expression between TCGA tumor samples and corresponding normal tissues. **(B)** Differences in PPME1 mRNA expression between tumor and normal tissues, integrating data from TCGA and GTEx datasets. **(C)** PPME1 mRNA expression in TCGA tumor samples compared to paired normal tissues. **(D)** Differences in PPME1 protein content in pan-carcinoma, including BRCA, COAD, OV, KIRC, UCEC, LUAD, PAAD, HNSC, GBM, and LIHC. *p < 0.05, **p < 0.01, ***p < 0.001.

**Figure 1A** reveals PPME1 mRNA expression is significantly higher in 14 cancer types, including bladder urothelial carcinoma (BLCA), breast invasive carcinoma (BRCA), cholangiocarcinoma (CHOL), colon adenocarcinoma (COAD), esophageal carcinoma (ESCA), head and neck squamous cell carcinoma (HNSC), kidney renal clear cell carcinoma (KIRC), liver hepatocellular carcinoma (LIHC), lung adenocarcinoma (LUAD), lung squamous cell carcinoma (LUSC), prostate adenocarcinoma (PRAD), rectum adenocarcinoma (READ), stomach adenocarcinoma (STAD), and uterine corpus endometrial carcinoma (UCEC). In contrast, PPME1 mRNA expression is significantly reduced in glioblastoma multiforme (GBM) and kidney chromophobe (KICH) tissues compared to normal tissues.

To evaluate the reliability of our findings, we compared PPME1 expression between normal tissues from GTEx and tumor tissues from TCGA. This analysis demonstrated a significant upregulation of PPME1 mRNA in 23 cancer types, including BLCA, BRCA, cervical squamous cell carcinoma and endocervical adenocarcinoma (CESC), CHOL, COAD, diffuse large B-cell lymphoma (DLBC), ESCA, GBM, HNSC, KIRC, kidney renal papillary cell carcinoma (KIRP), brain lower-grade glioma (LGG), LIHC, LUAD, LUSC, ovarian serous cystadenocarcinoma (OV), pancreatic adenocarcinoma (PAAD), PRAD, READ, STAD, thymoma (THYM), UCEC, and uterine carcinosarcoma (UCS). In contrast, PPME1 mRNA was significantly downregulated in acute myeloid leukemia (LAML), skin cutaneous melanoma (SKCM), and testicular germ cell tumors (TGCT) relative to healthy tissues (*p*<0.05; **Figure 1B**).

In a study of 23 different malignant tumor types, PPME1 mRNA expression was significantly higher in BLCA, BRCA, CHOL, COAD, ESCA, HNSC, KIRC, LIHC, LUAD, LUSC, PRAD, READ, STAD, THCA, and UCEC compared to normal tissues, but significantly lower in KICH (*p*<0.05; **Figure 1C**).

In a similar vein, analysis using the UALCAN database corroborated that PPME1 protein levels are overexpressed in BRCA, COAD, KIRC, lung cancer, PAAD, HNSC, and LIHC, while they are underexpressed in GBM (*p*<0.05; **Figure 1D**). We assessed PPME1 protein expression in normal tissues and in multiple cancers—including BLCA, BRCA, LIHC, CHOL, COAD, READ, GBM, HNSC, KIRC, PRAD, OV, UCEC, LUAD, LUSC, PAAD, and SKCM—using the HPA database. The results at the protein level were in agreement with the earlier mRNA expression findings (**Supplementary Figure 1**).

### The connection between PPME1 and clinical characteristics

**Figure 2:**
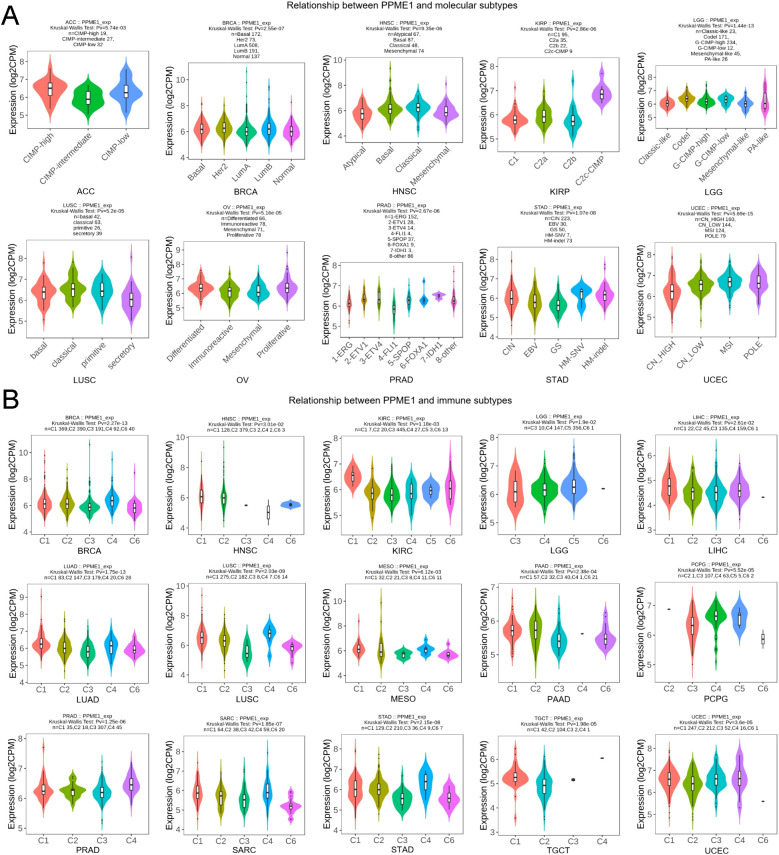
Correlation of PPME1 expression with different tumour subtypes. **(A)** Associations between molecular subtypes and PPME1 expression in diverse TCGA tumors, comprising ACC, BRCA, HNSC, KIRP, LGG, LUSC, OV, PRAD, STAD, and UCEC. **(B)** Relationships between immune subtypes and PPME1 expression across various TCGA tumors, encompassing BRCA, HNSC, KIRC, LGG, LIHC, LUAD, LUSC, MESO, PAAD, PCPG, PRAD, SARC, STAD, TGCT, and UCEC.

We thoroughly examined the relationship between PPME1 expression and the immune and molecular subtypes across various cancers. Our findings indicated that PPME1 expression levels exhibited variability across ten distinct cancer subtypes characterized by unique molecular profiles. Elevated PPME1 expression was notably observed in specific cancer subtypes: CIMP-high in ACC, Her2 in BRCA, classical in HNSC and LUSC, C2c-CIMP in KIRP, codel in LGG, proliferative in OV, 7-IDH1 in PRAD, HM-SNV in STAD, and MSI in UCEC (**Figure 2A**).

Our analysis identified six immune subtypes of tumors: C1 (wound healing), C2 (IFN-γ dominant), C3 (inflammatory), C4 (lymphocyte depleted), C5 (immunologically quiet), and C6 (TGF-β dominant). PPME1 expression showed a significant correlation with these subtypes across 15 cancers, including BRCA, HNSC, KIRC, LGG, LIHC, LUAD, LUSC, MESO, PAAD, PCPG, PRAD, SARC, STAD, TGCT, and UCEC (**Figure 2B**). Among the subtypes, C3 showed the least PPME1 expression, whereas C1 and C4 showed the most.

### PPME1’s significance in cancer prognosis and diagnosis

**Figure 3:**
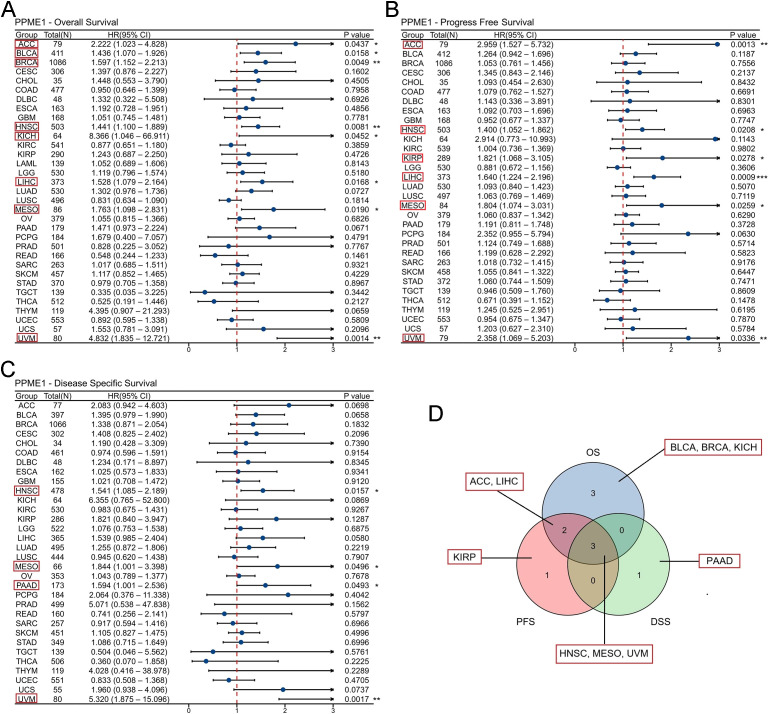
Association between PPME1 expression and prognosis in cancer patients. **(A)** Association between PPME1 expression and overall survival (OS) in cancer patients. **(B)** Association between PPME1 expression and progression-free survival (PFS) in cancer patients. **(C)** Association between PPME1 expression and disease-specific survival (DSS) in cancer patients. **(D)** The Venn diagram shows the intersection of OS, DSS, and PFS for different cancers. *p < 0.05, **p < 0.01, ***p < 0.001.

Samples were split into high- and low-expression groups according to the median PPME1 level to explore the connection between PPME1 expression and patient survival. Cox regression revealed that high PPME1 expression negatively impacted overall survival (OS) in several cancers, including ACC, BLCA, BRCA, HNSC, KICH, LIHC, MESO, and UVM (**Figure 3A****;****Supplementary Figure 2A**). High PPME1 was also a risk factor for progression-free survival (PFS) in ACC, HNSC, KIRP, LIHC, MESO, and UVM (**Figure 3B**; **Supplementary Figure 2B**), and for disease-specific survival (DSS) in HNSC, MESO, PAAD, and UVM (**Figure 3C**; **Supplementary Figure 2C**). In summary, our findings suggest that elevated PPME1 expression is a prognostic risk factor for poor outcomes in patients with ten types of cancer, including ACC, BLCA, BRCA, HNSC, KICH, KIRP, LIHC, MESO, PAAD and UVM (**Figure 3D**).

**Figure 4:**
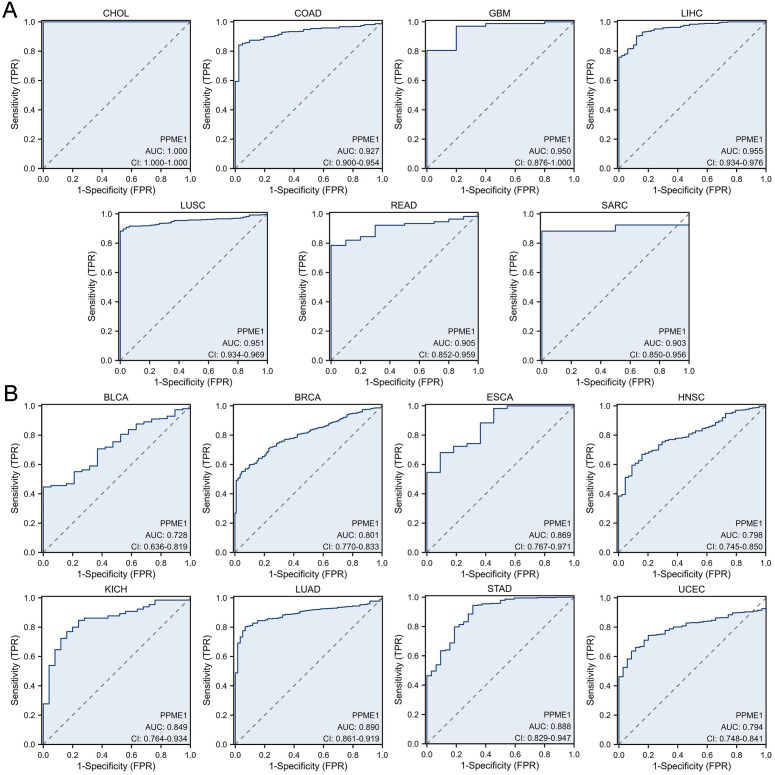
Receiver operating characteristic (ROC) curve for PPME1 expression in pan-cancer. **(A)** PPME1 expression in cancers with good diagnostic value (AUC>0.9), including CHOL (tumor N = 35, normal N = 9), COAD (tumor N = 480, normal N = 41), GBM (tumor N = 169, normal N = 5), LIHC (tumor N = 374, normal N = 50), LUSC (tumor N = 502, normal N = 49), READ (tumor N = 167, normal N = 10), and SARC (tumor N = 263, normal N = 2). **(B)** PPME1 expression in cancers with some diagnostic value (0.9>AUC>0.7), including BLCA (tumor N = 412, normal N = 19), BRCA (tumor N = 1113, normal N = 113), ESCA (tumor N = 163, normal N = 11), HNSC (tumor N = 504, normal N = 44), KICH (tumor N = 65, normal N = 25), LUAD (tumor N = 539, normal N = 59), STAD (tumor N = 375, normal N = 32), and UCEC (tumor N = 554, normal N = 35).

Subsequently, we evaluated the diagnostic efficacy of PPME1 across various cancer types. The analysis of the ROC curve revealed that PPME1 demonstrated outstanding diagnostic performance, with an AUC exceeding 0.9 in several cancers, including CHOL (1.000), COAD (0.927), GBM (0.950), LIHC (0.955), LUSC (0.951), READ (0.905), and SARC (0.903) (**Figure 4A**). In contrast, PPME1 exhibited moderate diagnostic performance, with an AUC exceeding 0.7, in other cancer types such as BLCA (0.728), BRCA (0.801), ESCA (0.869), HNSC (0.798), KICH (0.849), LUAD (0.890), STAD (0.888), and UCEC (0.794) (**Figure 4B**). It should be noted that these analyses are preliminary and exploratory, aiming to validate the differential expression pattern of PPME1 between tumor and normal tissues, rather than to support its use as a standalone diagnostic marker for clinical screening. Tissue biopsy-dependent detection does not fully mimic real-world non-invasive diagnostic scenarios.

Higher PPME1 expression is linked to worse prognosis in multiple cancers and is moderately to strongly effective in differentiating tumors from normal tissues in most malignancies.

### PPME1 independently influences the prognosis of certain cancers

We examined eight cancer types using regression analysis to identify factors affecting patient OS. For ACC, the primary therapy outcome is the sole prognostic factor (**Supplementary Table 2A**). In BLCA, both primary therapy outcome and high PPME1 expression are key (**Supplementary Table 2B**). BRCA’s factors include N stage, M stage, age over 60, and high PPME1 expression (**Supplementary Table 2C**). HNSC is influenced by T stage, N stage, and high PPME1 expression (**Supplementary Table 2D**). KICH and LIHC both show T stage and elevated PPME1 expression as factors (**Supplementary Tables 2E, F**). In MESO, only PPME1 expression was analyzed, so no independent factors were identified (**Supplementary Table 2G**). For UVM, Clinical M stage and high PPME1 expression are crucial (**Supplementary Table 2H**).

**Figure 5:**
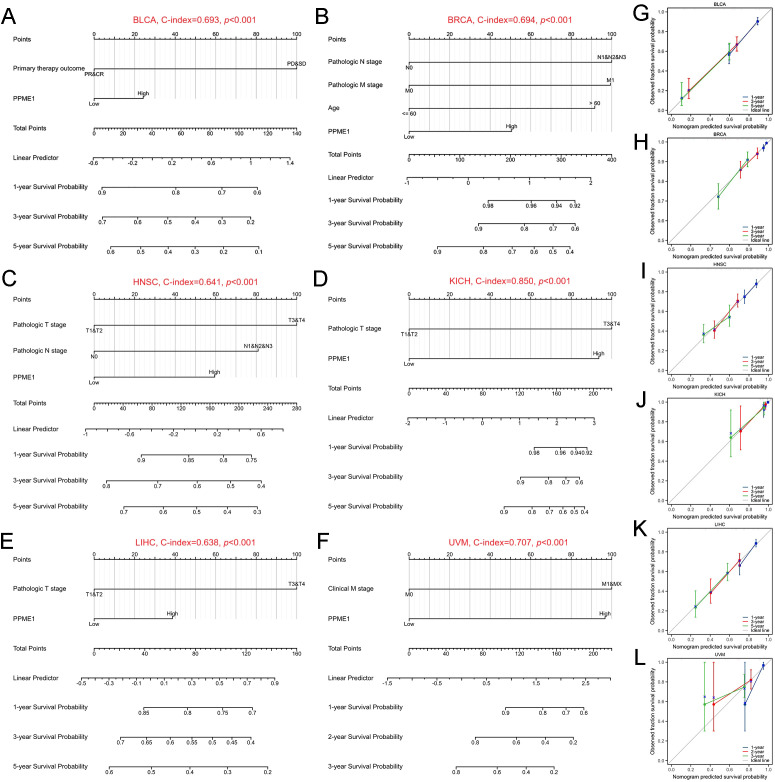
Nomograms and calibration curves predicting patient OS in six cancers. Nomograms for BLCA **(A)**, BRCA **(B)**, HNSC **(C)**, KICH **(D)**, LIHC **(E)**, and UVM **(F)**. Calibration curves for BLCA **(G)**, BRCA **(H)**, HNSC **(I)**, KICH **(J)**, LIHC **(K)**, and UVM **(L)**. The horizontal and vertical coordinates represent the model-predicted and observed survival probability, respectively. A closer alignment with the ideal line indicates better model performance.

Significant variables (p < 0.05) identified in the univariate Cox regression analysis were later used to build prognostic nomograms and evaluate their calibration. The resulting C-index values for each cancer type were as follows: BLCA (C-index=0.693, **Figure 5A**), BRCA (C-index=0.694, **Figure 5B**), HNSC (C-index=0.641, **Figure 5C**), KICH (C-index=0.850, **Figure 5D**), LIHC (C-index=0.638, **Figure 5E**), and UVM (C-index=0.707, **Figure 5F**).

For ACC and MESO, only one independent prognostic factor was identified, preventing the creation of a nomogram. Calibration curves for each nomogram showed high accuracy, closely aligning with the ideal line for all cancer types mentioned (**Figures 5G–L**). This suggests that PPME1 could be a dependable independent predictor of outcomes for patients with these tumors.

### Analysis of genetic variation in PPME1

**Figure 6:**
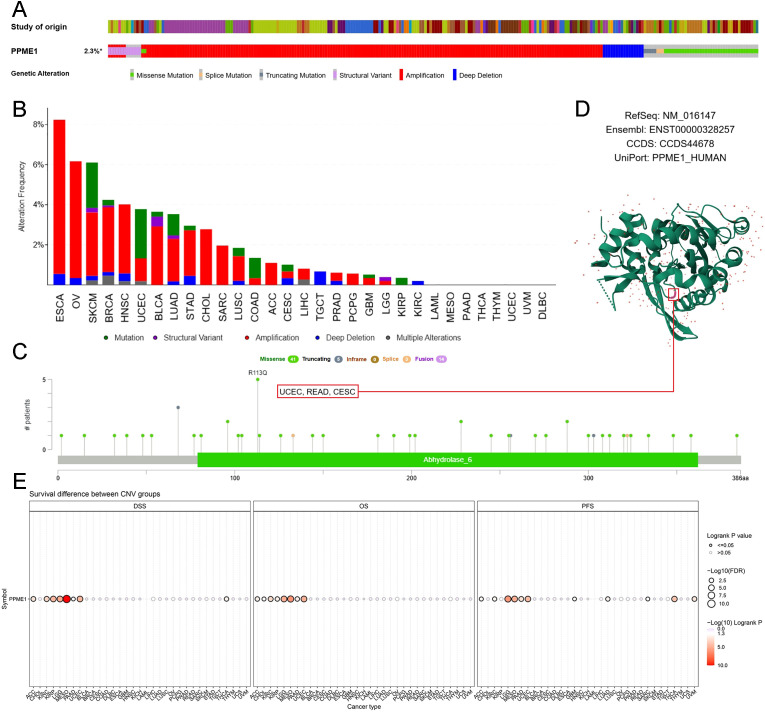
Mutated features of PPME1 in various tumors. **(A)** Overview of PPME1 expression alterations in different tumors. **(B)** Frequency distribution of mutation types. **(C)** Mutation sites in the PPME1 amino acid sequences. **(D)** Visualization of select PPME1 mutations on the 3D protein structure. **(E)** Association between CNV in PPME1 and cancer patient prognosis.

Cancer involves various genetic changes, some of which could be targeted for therapy (24). We studied these changes to see if PPME1 could be a therapeutic target. In 10,528 samples, 242 (2.3%) had PPME1 mutations, with amplification being the most common CNV alteration, followed by missense mutations and deep deletions (**Figure 6A**). The highest mutation frequencies were found in ESCA (8.24%), OV (6.16%), SKCM (6.11%), BRCA (4.24%), and HNSC (4.02%) (**Figure 6B**). The Abhydrolase_6 domain had frequent mutations, mainly R113Q, occurring twice in UCEC and READ, and once in CESC (**Figure 6C**). We also visualized these mutations in the 3D structure and sequence of PPME1 (**Figure 6D**). CNV in PPME1 was identified as a prognostic risk factor in ACC, KIRC, KIRP, LGG, MESO, PAAD, UCEC, and THCA (**Figure 6E**).

**Figure 7:**
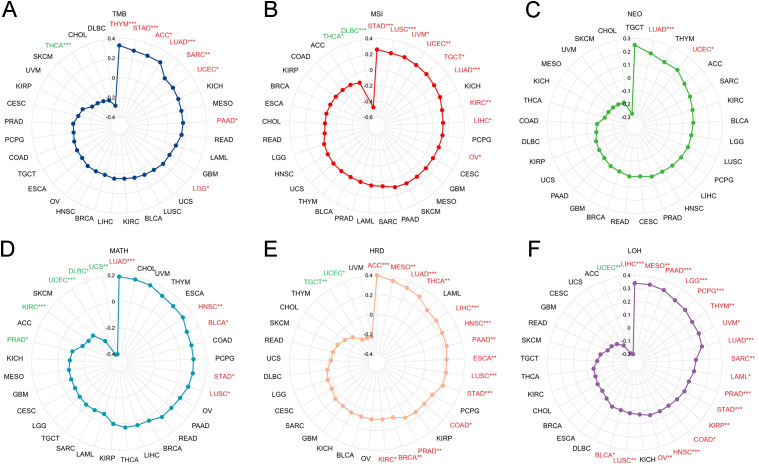
The relationship between PPME1 and various genomic heterogeneity scores in pan-cancer, including TMB **(A)**, MSI **(B)**, NEO **(C)**, MATH **(D)**, HRD **(E)**, and LOH **(F)**. *p < 0.05, **p < 0.01, ***p < 0.001. The associations between PPME1 expression and tumour heterogeneity scores were analyzed via Spearman’s correlation.

Genomic heterogeneity influences prognosis and treatment outcomes, and the correlation of PPME1 with six indicators is evaluated. PPME1 shows a positive correlation with tumor mutational burden (TMB) in THYM, STAD, ACC, LUAD, SARC, UCEC, PAAD, and LGG, but a negative one in THCA (**Figure 7A**). It is positively correlated with microsatellite instability (MSI) in STAD, LUSC, UVM, UCEC, TGCT, LUAD, KIRC, LIHC, and OV, and negatively in THCA and DLBC (**Figure 7B**). PPME1 correlates positively with neoantigen (NEO) only in LUAD and UCEC (**Figure 7C**). It shows a positive correlation with mutant-allele tumor heterogeneity (MATH) in LUAD, HNSC, BLCA, STAD, and LUSC, and a negative one in PRAD, KIRC, UCEC, DLBC, and UCS (**Figure 7D**). PPME1 is positively correlated with homologous recombination deficiency (HRD) in ACC, MESO, LUAD, THCA, LIHC, HNSC, PAAD, ESCA, LUSC, STAD, COAD, PRAD, BRCA, and KIRC, but negatively in TGCT and UCEC (**Figure 7E**). Lastly, PPME1 is positively correlated with loss of heterozygosity (LOH) in LIHC, MESO, PAAD, LGG, PCPG, THYM, UVM, LUAD, SARC, LAML, PRAD, STAD, KIRP, COAD, HNSC, OV, LUSC, and BLCA, and negatively only in UCEC (**Figure 7F**).

### Relationship between PPME1 expression and immunity

**Figure 8:**
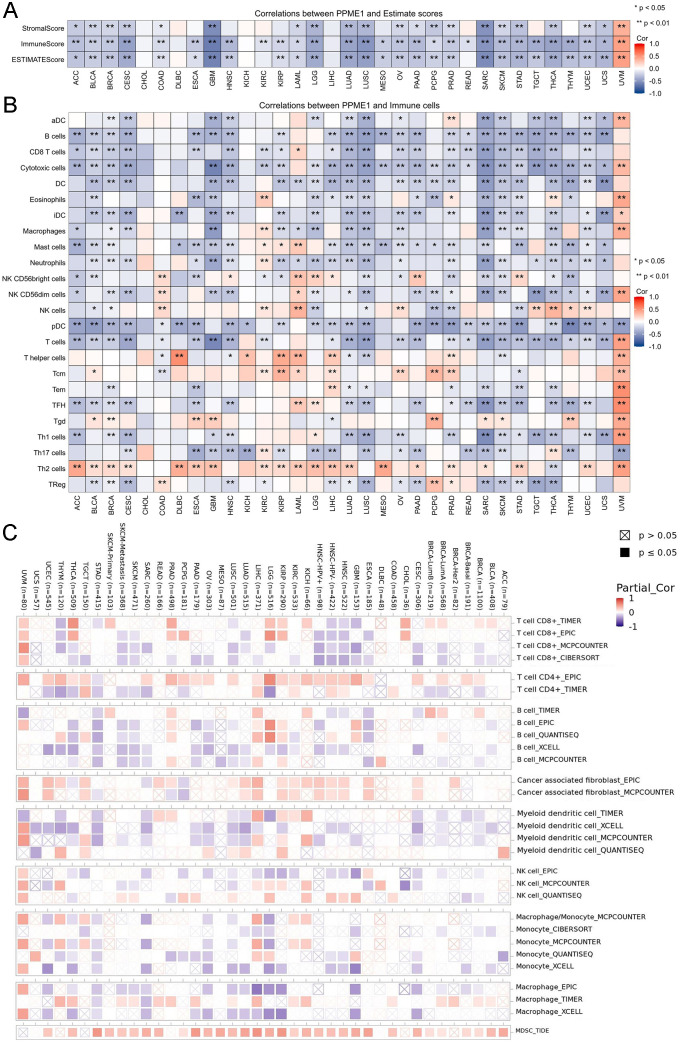
The link between PPME1 and immune infiltration scores and levels across various cancers. **(A)** Correlation between PPME1 expression and StromalScore, ImmuneScore, and ESTIMATEScore in 33 cancers. **(B)** Correlation between PPME1 expression and immune infiltration using the ssGSEA algorithm. **(C)** Correlation analysis of PPME1 expression with multiple immune cell infiltrates in pan-carcinogenesis. *p < 0.05, **p < 0.01, ***p < 0.001.

The ESTIMATE algorithm was then applied to evaluate the relationship of PPME1 expression with stromal and immune scores in multiple cancer types. PPME1 expression showed a negative correlation with stromal, immune, and ESTIMATE scores in ACC, BLCA, BRCA, CESC, COAD, GBM, LAML, LGG, LUAD, LUSC, OV, PAAD, PCPG, PRAD, SARC, SKCM, STAD, THCA, UCEC, and UCS. In UVM, however, PPME1 expression was positively correlated with these three scores (**Figure 8A**).

Initially, we examined PPME1 expression levels in various single cells across multiple cancer types and discovered that PPME1 is primarily located in cancer cells, exhausted T (Tex) cells, fibroblasts, endothelial cells, and Tproliferative cells (**Supplementary Figure 3**). Tumor-infiltrating immune cells (TIICs) play a crucial role in the tumor microenvironment and cancer progression. The ssGSEA algorithm was employed to analyze the connection between PPME1 expression and the infiltration levels of 24 unique immune cell types. The resulting heatmap showed that PPME1 expression is predominantly negatively correlated with B cells, CD8+ T cells, cytotoxic cells, plasmacytoid dendritic cells (pDCs), and T cells. Conversely, PPME1 expression exhibited a positive correlation with tumor-promoting T helper 2 (Th2) cells (**Figure 8B**). Further analysis using Timer 2.0 confirmed these findings and revealed that PPME1 expression is also positively correlated with CAFs and MDSCs infiltration in most tumors (**Figure 8C**).

### Functional enrichment analysis of PPME1

**Figure 9:**
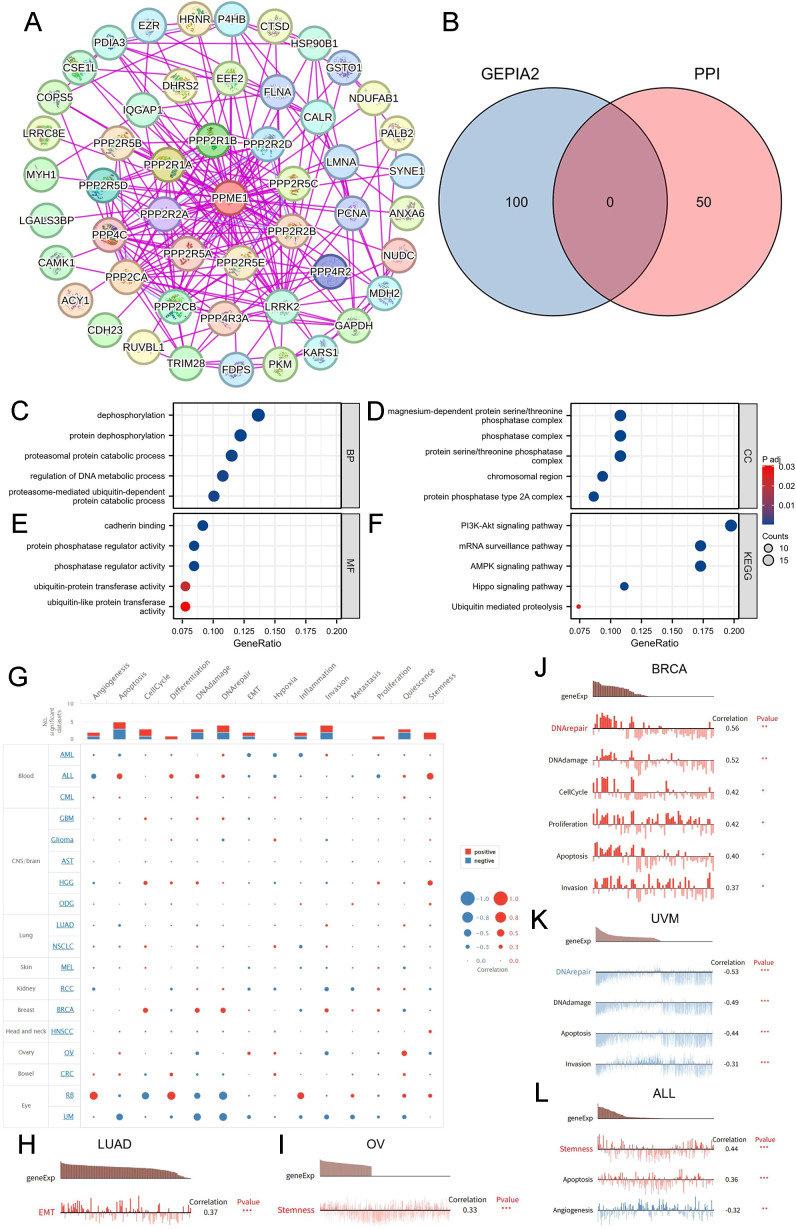
PPME1-related genes, interacting proteins, and functional enrichment analysis. **(A)** Protein-protein interaction (PPI) network for PPME1. **(B)** Venn diagram showing the intersection of PPME1-binding and interacting genes after selection. GO analysis includes biological processes **(C)**, cellular components **(D)**, molecular functions **(E)**, and KEGG pathways **(F)**. **(G)** The interactive bubble chart of connections between PPME1 and functional status in the CancerSEA database. The relationships between PPME1 expression and functional statuses in LUAD **(H)**, OV **(I)**, BRCA **(J)**, UVM **(K)**, and ALL **(L)**. *p < 0.05, **p < 0.01, ***p < 0.001.

To understand the involvement of PPME1 in cancer, we executed enrichment analyses and constructed a protein-protein interaction network using 50 proteins that bind to PPME1, as identified in the STRING database (**Figure 9A**). The genes correlated with PPME1 expression were also identified from the GEPIA2 resource (**Supplementary Table 3**). The Venn diagram revealed no overlapping genes (**Figure 9B**). GO and KEGG enrichment analyses of PPME1-related genes identified 83 significant categories, including 49 biological processes (BP), 23 cellular components (CC), 11 molecular functions (MF), and 15 KEGG pathways (**Supplementary Table 4**). In cancer-related GO-BP terms, PPME1 was linked to protein dephosphorylation, DNA metabolic regulation, and proteasomal protein catabolism (**Figure 9C**). GO-CC analysis showed enrichment in protein phosphatase complexes and chromosomal regions (**Figure 9D**), while GO-MF analysis suggested involvement in cadherin binding, protein phosphatase regulation, and ubiquitin-like protein transferase activity (**Figure 9E**). KEGG analysis indicated PPME1’s potential role in the PI3K-Akt, AMPK, mRNA surveillance, Hippo signaling pathways, and ubiquitin-mediated proteolysis (**Figure 9F**).

Subsequently, we investigated the influence of PPME1 on the functional state of cancer cells at the single-cell level using the CancerSEA database (**Figure 9G**). Our analysis revealed that PPME1 exhibits a positive correlation with epithelial-mesenchymal transition (EMT) in LUAD (**Figure 9H**), and with stemness in OV (**Figure 9I**). In BRCA, PPME1 is positively linked to DNA repair, damage, cell cycle, proliferation, apoptosis, and invasion (**Figure 9J**), while in UVM, it is negatively associated with DNA repair, damage, apoptosis, and invasion (**Figure 9K**). Furthermore, in ALL, PPME1 is positively correlated with stemness and apoptosis, but negatively correlated with angiogenesis (**Figure 9L**).

**Figure 10:**
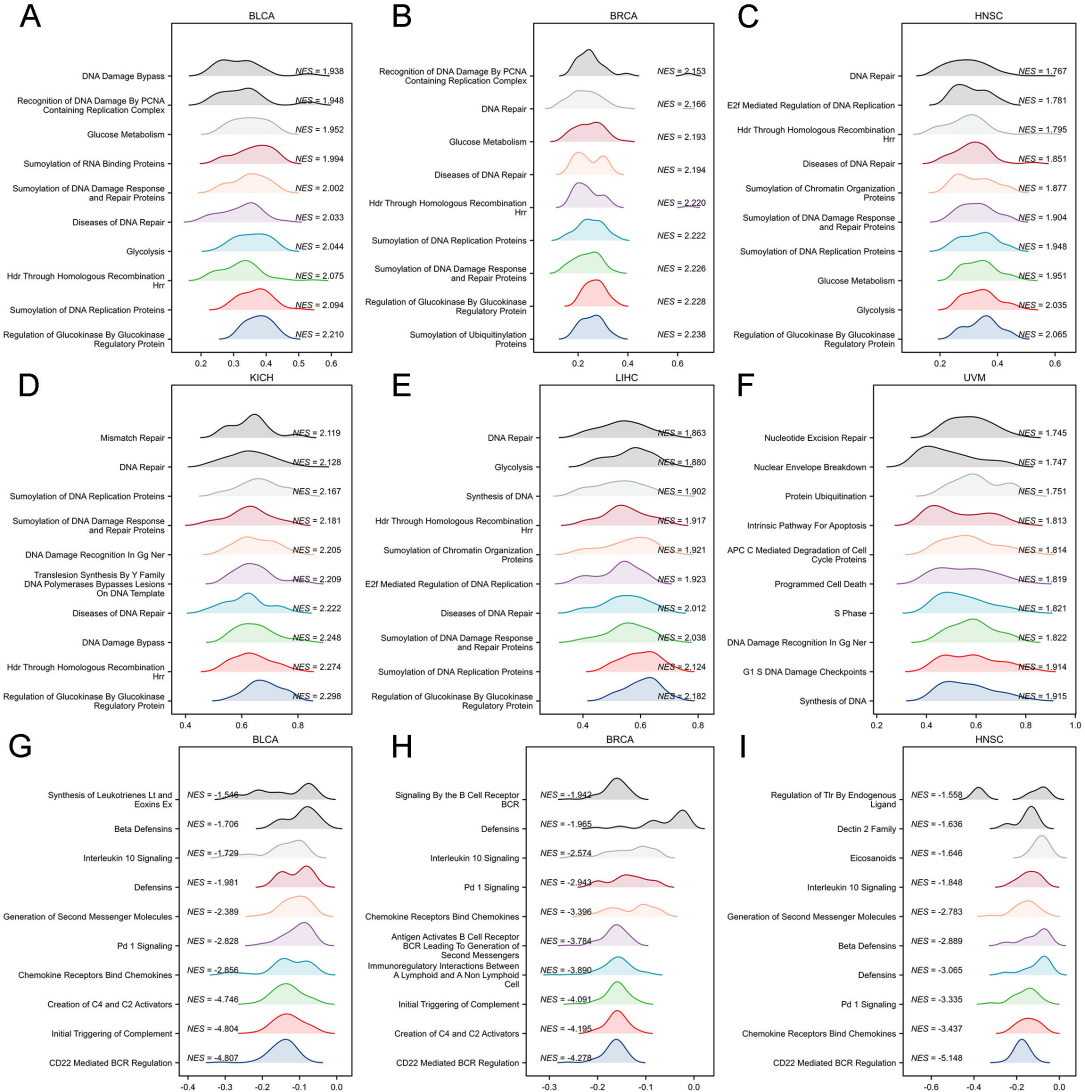
GSEA functional enrichment analysis of PPME1 in six types of cancer. In BLCA **(A)**, BRCA **(B)**, HNSC **(C)**, KICH **(D)**, LIHC **(E)**, and UVM **(F)**, the first 10 pathways are positively correlated with PPME1 expression. In BLCA **(G)**, BRCA **(H)**, and HNSC **(I)**, the first 10 pathways are negatively correlated with PPME1 expression. The remaining cancers were not enriched in negatively correlated pathways and therefore are not shown.

We performed GSEA using the Reactome pathway database to identify pathways associated with PPME1 in six cancer types: BLCA, BRCA, HNSC, KICH, LIHC, and UVM. Our results in **Figures 10A–F** show that genes positively associated with PPME1 are enriched in pathways related to DNA damage and repair, glucose metabolism, glycolysis, SUMOylation, cell cycle, and apoptosis. Conversely, genes negatively associated with PPME1 are enriched in immune-related pathways (such as BCR regulation, complement cascade, PD-1 signaling, interferon, and lymphoid/non-lymphoid cell interactions) and various metabolism processes (including fatty acids, bile salts, urea, and amino acids) (**Figures 10G–I**).

### Expression and functional validation of PPME1 in BRCA

**Figure 11:**
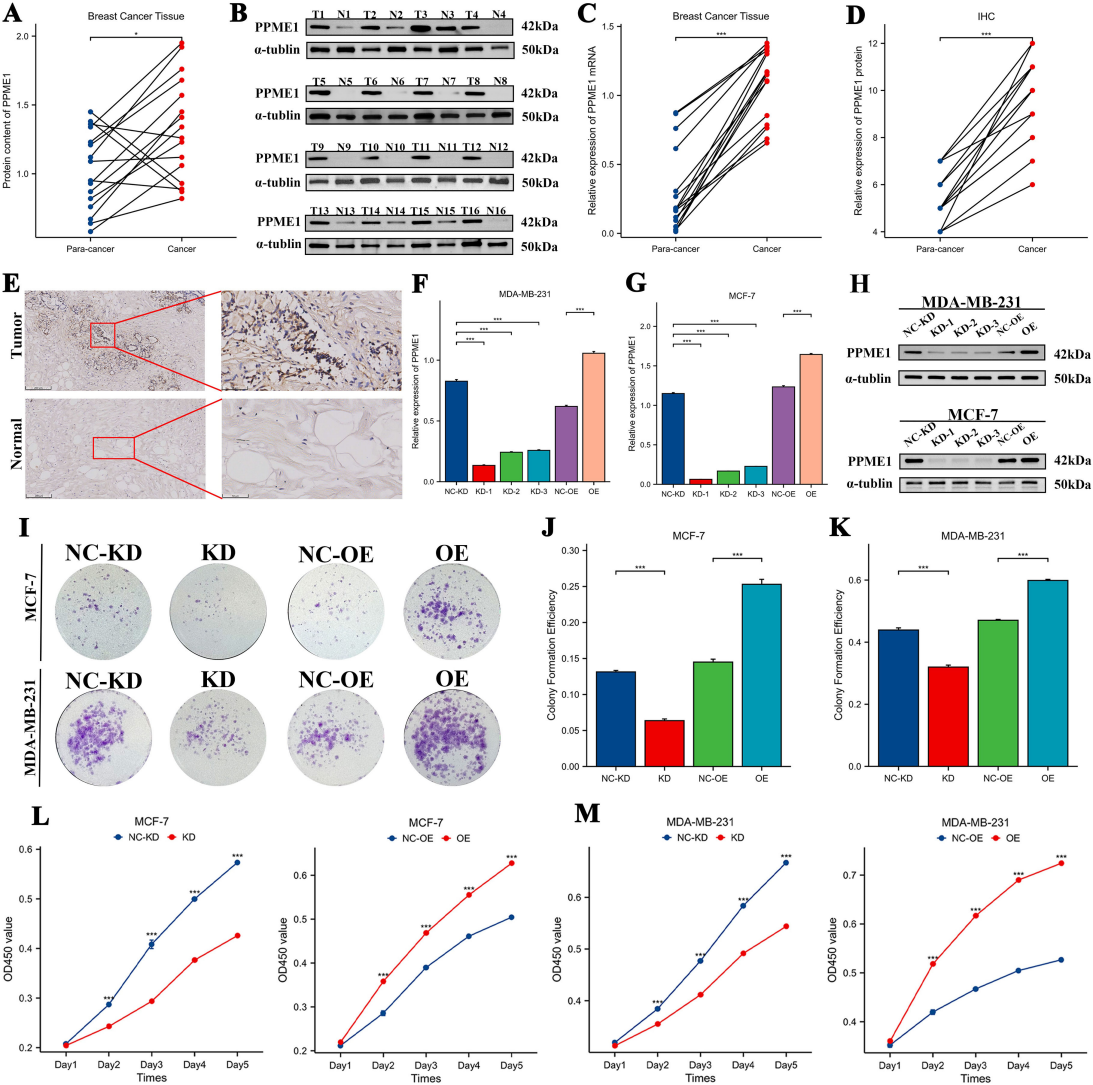
Verify PPME1 mRNA expression and protein levels in clinical samples, and assess how PPME1 regulation affects breast cancer cell proliferation. The mRNA expression levels and protein content differences of PPME1 were verified using 16 pairs of breast cancer tissues, including Western blot experiments **(A, B)**, RT-qPCR experiments **(C)**, and immunohistochemistry experiments **(D, E)**. **(F-H)** RT-qPCR and WB experiments verified the knockdown and overexpression efficiency in MDA-MB-231 and MCF-7 cells. Changes in cell proliferation ability were detected by colony formation assay **(I-K)** and CCK-8 assay **(L, M)**. *p < 0.05, **p < 0.01, ***p < 0.001.

**Figure 12:**
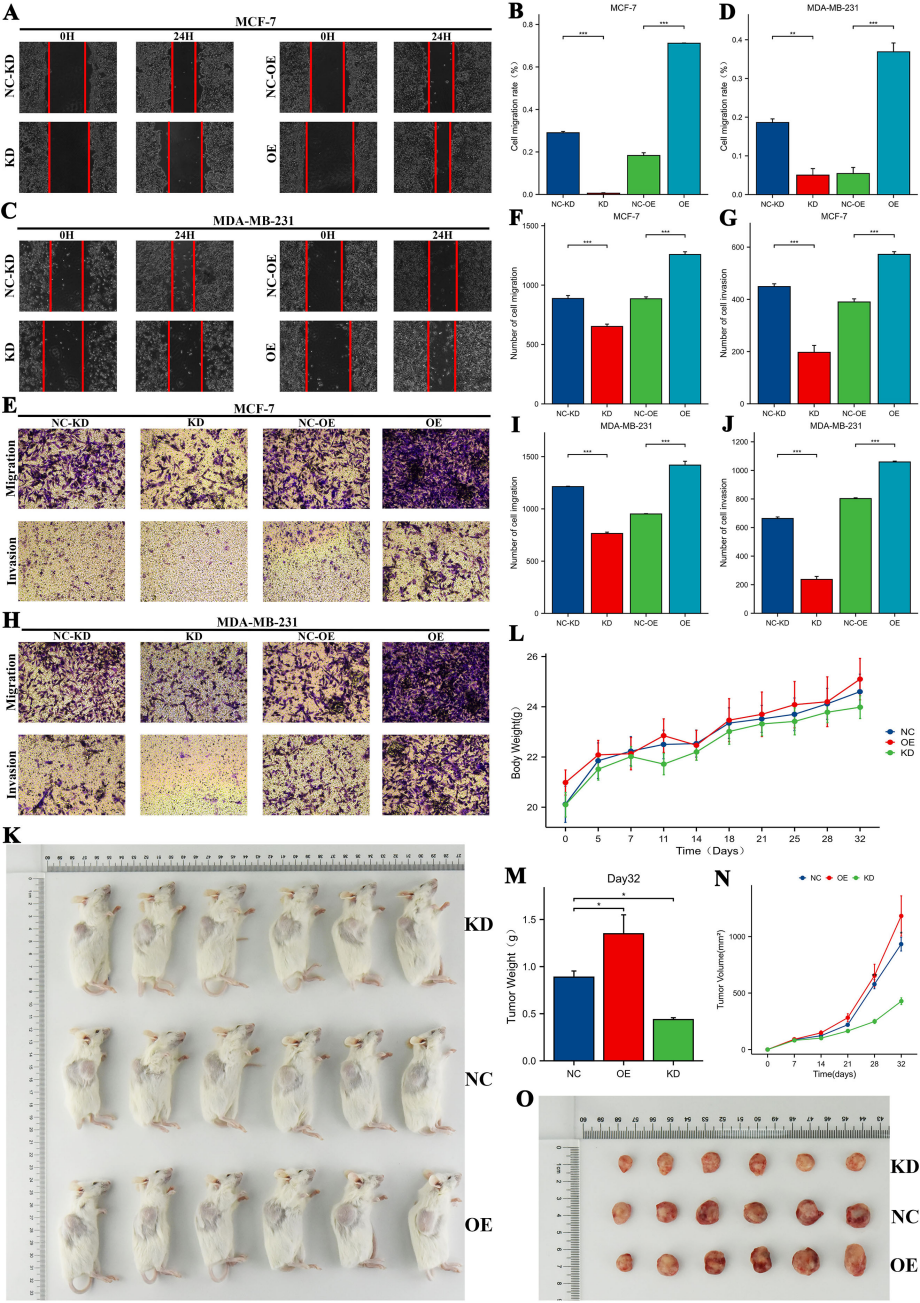
Assess how PPME1 regulation influences breast cancer cell invasion and migration, and examine its effect on breast tumor growth in animal models. The effects of PPME1 regulation on the invasion and migration abilities of MDA-MB-231 and MCF-7 cells were examined using wound healing assays **(A-D)**, Transwell migration assays, and Transwell invasion assays **(E-J)**. Gross images of xenograft mouse models **(K)**. Body weight changes of xenograft mouse models **(L)**. Tumour weight at day 32 **(M)**. Tumour growth curve in xenograft mouse models **(N)**. Tumour images at day 32 **(O)**. *p < 0.05, **p < 0.01, ***p < 0.001.

We analyzed PPME1 mRNA and protein levels in 16 BRCA samples and adjacent tissues. Western blot (**Figures 11A, B**), RT-qPCR (**Figure 11C**), and immunohistochemistry (**Figures 11D, E**) confirmed PPME1 overexpression in BRCA, indicating its potential role in tumor development. To investigate how different PPME1 expression levels affect breast cancer malignancy, we generated MCF-7 and MDA-MB-231 cell lines with PPME1 knockdown and overexpression. RT-qPCR and Western blotting confirmed these genetic modifications (**Figures 11F–H**). Results from colony formation (**Figures 11I–K**) and CCK-8 assays (**Figures 11L, M**) revealed that PPME1 knockdown inhibited the proliferation of breast cancer cell lines, whereas overexpression facilitated proliferation. Furthermore, wound healing (**Figures 12A–D**) and Transwell assays (**Figures 12E–J**) demonstrated that PPME1 knockdown significantly reduced the migration and invasion capabilities of the breast cancer cell lines, in contrast to PPME1 overexpression, which enhanced these abilities. *In vivo* experiments further corroborated these findings, showing that PPME1 knockdown suppressed tumor growth in a subcutaneous xenograft model, whereas overexpression accelerated tumor growth (**Figures 12K–O**). In conclusion, the experimental findings indicate that PPME1 is significantly overexpressed in breast cancer, promoting the proliferation, migration, and invasion of cancer cells.

## Discussion

This groundbreaking study examines PPME1’s expression, role, and genetic mechanisms across multiple cancers to find new treatment targets. PPME1 is highly overexpressed in most cancers compared to normal tissues and is associated with poor patient outcomes, particularly in ACC, BLCA, BRCA, HNSC, KICH, LIHC, LUAD, MESO, PAAD, and UVM. Research already indicates that PPME1 is highly expressed in pancreatic cancer and poses a risk for poor prognosis in PAAD patients, which is in agreement with our results (7). Guo et al. also linked high PPME1 expression to poor prognosis in THCA patients (9), a difference we noted but could not confirm in terms of prognostic value, necessitating further clinical validation. Kaur et al. reported longer OS and PFS in COAD patients with high PPME1 expression (25), a finding we observed only in expression levels, suggesting a discrepancy in its prognostic significance that also requires additional clinical validation. PPME1 expression varies across cancer subtypes, being highest in C1 and C4 immune subtypes and lowest in C3. Notably, C3 has the best survival prognosis, while C4 has the worst, aligning with previous research findings (26).

This study conducted an in-depth investigation into the diagnostic and prognostic significance of PPME1 across various types of cancer. Elevated PPME1 expression was independently associated with poor prognosis in patients with BLCA, BRCA, HNSC, KICH, LIHC, and UVM. Furthermore, predictive models integrating PPME1 expression and clinical data were developed, demonstrating high stability and predictive accuracy (C-index approaching 0.7), thereby enhancing their potential clinical utility. However, further comprehensive validation is warranted. In contrast to the findings of Junak et al., who utilized ten gene features, including PPME1, to predict radiation exposure-related childhood THCA (27), this study did not observe such an association, suggesting that this phenomenon may be specific to post-radiation exposure contexts. Additionally, PPME1 exhibited strong diagnostic performance in CHOL, LIHC, LUSC, and READ, with an AUC exceeding 0.9. Nevertheless, given that PPME1 is not a secreted protein, its translational potential requires further evaluation, and the development of more precise detection methodologies may be necessary to facilitate clinical translation in the future. Regarding the clinical relevance of PPME1, given that it is not a secreted protein, its potential as a non-invasive diagnostic biomarker is limited. Instead, PPME1 shows greater promise as a tissue-based prognostic indicator. The correlation between high PPME1 expression and poor prognosis in breast cancer and other cancer types suggests that it can be used to stratify patient risk and guide individualized treatment strategies. Additionally, PPME1 may serve as an auxiliary pathological marker to improve the diagnostic accuracy of ambiguous tumor samples when combined with other established markers.

Epigenomic dysregulation can lead to abnormal transcription, impacting tumor immunogenicity and immune responses, and contributing to cancer progression (28, 29). Our study identified PPME1 gene mutations in most tumors, correlating with increased expression levels. Specifically, PPME1 amplification mutations are linked to higher expression in ACC, KIRP, MESO, and PAAD cancers, potentially influencing patient prognosis. Li et al. found similar PPME1 amplification in STAD and lung cancer, associating it with poor prognosis and suggesting it as a therapeutic target (30). Although we didn’t observe this, their findings complement our research. Substantial evidence indicates that numerous predictive biomarkers, including TMB, MSI, and NEO, are capable of forecasting the antitumor efficacy of immune checkpoint inhibitors (ICIs) (31). High TMB and MSI levels are typically linked to better ICI therapy responses and prognosis (32, 33). This study explored the relationship between PPME1 and TMB, NEO, and MSI, finding a positive correlation in STAD, LUAD, and UCEC. This suggests that elevated PPME1 expression may serve as an indicator of suitability for immunotherapy in patients with these malignancies. Furthermore, measures such as MATH, HRD, and LOH are crucial indicators of genomic heterogeneity and are linked to tumor prognosis and immunotherapy outcomes (34). The study also demonstrates a significant association between PPME1 and MATH, HRD, and LOH across various cancers. These findings imply that PPME1 may influence the tumor microenvironment by affecting genomic stability, thereby modulating the response to immunotherapy. Copy number amplification typically leads to a dose-dependent elevation of gene transcription, which may directly contribute to the increased PPME1 expression levels detected in tumor tissues and subsequent pro-tumorigenic effects, including enhanced cell proliferation, migration and invasion. Collectively, these findings indicate that genetic alterations of PPME1 in cancers are dominated by copy number amplification rather than somatic mutation.

TME encompasses a diverse array of infiltrating immune cells, among which CAFs play a pivotal role by actively facilitating cancer progression through intricate interactions with other cellular constituents of the TME (35). MDSCs shield cancer cells from immune-mediated destruction and immunotherapy, thereby facilitating tumor progression (36). This study finds that PPME1 expression is positively linked to CAFs and MDSCs, which aid in immune evasion in the TME. Conversely, PPME1 expression is negatively associated with most antitumor immune cells, such as B cells, CD8+ T cells, cytotoxic cells, pDCs, and T cells. Additionally, our observations reveal that the correlation between PPME1 and pro-tumor Th2 cells is significantly stronger than that with antitumor T helper 1 (Th1) cells, suggesting that elevated PPME1 expression may induce a shift in the immune response from an antitumor to a pro-tumor orientation (37). Our bioinformatic analyses using TIMER2.0 and ESTIMATE datasets predicted a negative correlation between PPME1 expression and cytotoxic T-cell infiltration in breast cancer. However, these findings are based on in silico data mining and require further experimental validation using clinical samples. Future studies are warranted to perform multiplex IHC on clinical breast cancer tissue specimens, which can simultaneously detect PPME1 expression and the abundance of cytotoxic T cells (e.g., CD8+ T cells) in the same tissue section. Such experiments will help to confirm the regulatory role of PPME1 in modulating the tumor immune microenvironment and explore the potential clinical utility of combining PPME1 expression with immune cell infiltration patterns for prognostic stratification and treatment guidance of breast cancer patients. Our *in vivo* subcutaneous xenograft experiments demonstrated that PPME1 knockdown significantly suppressed tumor growth in NCG mice. It is critical to note that NCG mice are a severely immunodeficient strain lacking functional T, B, and NK cells; thus, the observed phenotype of reduced tumor growth reflects the intrinsic proliferative capacity of breast cancer cells rather than immune modulation effects. While our pan-cancer bioinformatic analyses suggested potential correlations between PPME1 expression and immune cell infiltration patterns (e.g., CD8+ T cell abundance), the immunodeficient nature of the NCG mouse model precludes validation of direct immunoregulatory functions of PPME1 *in vivo*. Future studies employing immune-competent mouse models or humanized xenograft models are warranted to elucidate the potential role of PPME1 in shaping the tumor immune microenvironment and its underlying mechanisms.

With the advancement of research into the molecular mechanisms underlying BRCA pathogenesis, molecular targeted therapy has emerged as a pivotal strategy in the treatment of BRCA. Current molecular targets for clinical BRCA therapy include HER2 (38), PARP (39), VEGF (40), TROP-2 (41), and PD-1/PD-L1 (42, 43), among others. Nevertheless, the limited efficacy of molecular targeted therapy in BRCA treatment underscores the necessity for the identification of additional biological targets. Our research, both *in vivo* and *in vitro*, indicates that the suppression of PPME1 leads to a significant reduction in migration, invasion, and tumor growth of breast cancer cells and tumors. The Transwell assay results demonstrate that PPME1 knockdown inhibits the migration and invasion potential of breast cancer cells *in vitro*, while the subcutaneous xenograft model data show that PPME1 silencing suppresses tumor growth and proliferation *in vivo*. Notably, the current study does not include the detection of distant metastatic nodules (e.g., lung or liver histology), and thus no conclusion regarding metastasis inhibition is drawn. PPME1 may function as a potential biomarker for targeted therapy in BRCA, according to these findings.

## Conclusion

This research introduces the first all-encompassing pan-cancer analysis of the oncogene PPME1, merging omics, prognostic, epigenetic, immune-related, and functional enrichment evaluations. We confirmed PPME1’s expression and function in breast cancer using various *in vivo* and *in vitro* methods. These findings enhance our understanding of PPME1’s oncogenic role and offer new insights for cancer immunotherapy strategies.

## Data Availability

The original contributions presented in the study are included in the article/**Supplementary Material**. Further inquiries can be directed to the corresponding authors.

## Glossary

PPME1: Protein Phosphatase Methylesterase 1
TCGA: The Cancer Genome Atlas
GEO: Gene Expression Omnibus
PP2A: Protein Phosphatase 2A
ROC: Receiver Operating Characteristic
AUC: Area Under Curve
CNV: Copy Number Variation
SNV: Single Nucleotide Variant
C-index: consistency index
CAFs: cancer-associated fibroblasts
NK: natural killer
MDSCs: myeloid-derived suppressor cells
NC-KD: knockdown with an empty vector
KD: knockdown
NC-OE: overexpression with a vector
OE: overexpression
IHC: immunohistochemical
HR: Hazard ratios
FDR: false discovery rate
BLCA: bladder urothelial carcinoma
BRCA: breast invasive carcinoma
CHOL: cholangiocarcinoma
COAD: colon adenocarcinoma
ESCA: esophageal carcinoma
HNSC: head and neck squamous cell carcinoma
KIRC: kidney renal clear cell carcinoma
LIHC: liver hepatocellular carcinoma
LUAD: lung adenocarcinoma
LUSC: lung squamous cell carcinoma
PRAD: prostate adenocarcinoma
READ: rectum adenocarcinoma
STAD: stomach adenocarcinoma
UCEC: uterine corpus endometrial carcinoma
GBM: glioblastoma multiforme
KICH: kidney chromophobe
CESC: cervical squamous cell carcinoma and endocervical adenocarcinoma
DLBC: diffuse large B-cell lymphoma
KIRP: kidney renal papillary cell carcinoma
LGG: brain lower-grade glioma
OV: ovarian serous cystadenocarcinoma
PAAD: pancreatic adenocarcinoma
THYM: thymoma
UCS: uterine carcinosarcoma
LAML: acute myeloid leukemia
SKCM: skin cutaneous melanoma
TGCT: testicular germ cell tumors
OS: overall survival
PFS: progression-free survival
DSS: disease-specific survival
TMB: Tumor mutation burden
MSI: Microsatellite instability
GTEx: Genotype-Tissue Expression databases
TMB: tumor mutational burden
MSI: microsatellite instability
NEO: neoantigen
LOH: loss of heterozygosity
MATH: mutant-allele tumor heterogeneity
HRD: homologous recombination deficiency
Tex: exhausted T
TIICs: Tumor-infiltrating immune cells
pDCs: plasmacytoid dendritic cells
Th2: tumor-promoting T helper 2
BP: biological processes
CC: cellular components
MF: molecular functions
EMT: epithelial-mesenchymal transition
ICIs: immune checkpoint inhibitors
Th1: T helper 1
