# Supplementary material for: The value of Protein Phosphatase Methylesterase 1 in diagnosis, prognosis and immunoregulation: from pan-cancer analysis to breast cancer verification
Source: Front Immunol. 2026 Mar 10;17:1770711. doi: 10.3389/fimmu.2026.1770711 (PMC13008989; doi:10.3389/fimmu.2026.1770711)
Supplement: Supplementary file 1 [file DataSheet1.pdf]

Supplementary materials for

The value of Protein Phosphatase Methylesterase 1 in diagnosis, prognosis and immunoregulation: from pan-cancer analysis to b reast cancer verification

Yiyang Wang[1], Yue Zhang[2], Yongxiang Li[1], Haotian Ma[1], Jiawei Zhao[1], Dilimulati Ism tula[1*], Chenming Guo[1*]

1 Department of Breast Surgery, Center of Digestive and Vascular, The First Affiliated Hospital of Xinjiang Medical University, Urumqi 830054, China;

2 Thyroid and Breast Surgery Department of the People's Hospital of Bayingolin Mon gol Autonomous Prefecture, Korla 841000, China

*Correspondence: Chenming Guo, gcm_xjmu@yeah.net; Dilimulati Ismtula, mlt0306@si na.com

Additional material for this article can be found in the Supplementary Graphics and T able Legend module.

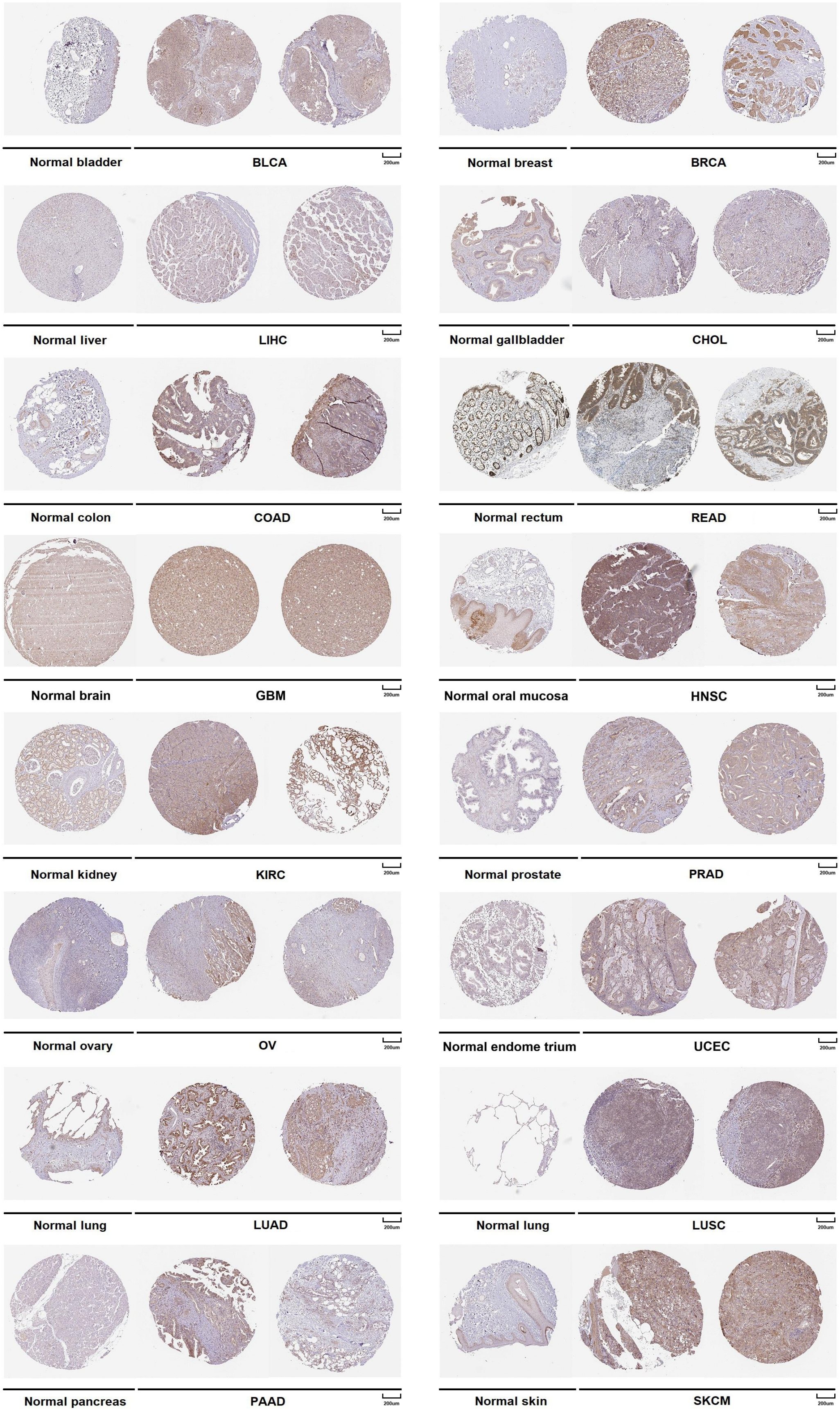

Supplementary Figure S1. Immunohistochemical Staining of 16 Normal Tissues and Tumor Tissues from the HPA Database. (Antibody: HPA043900, 100x magnification)

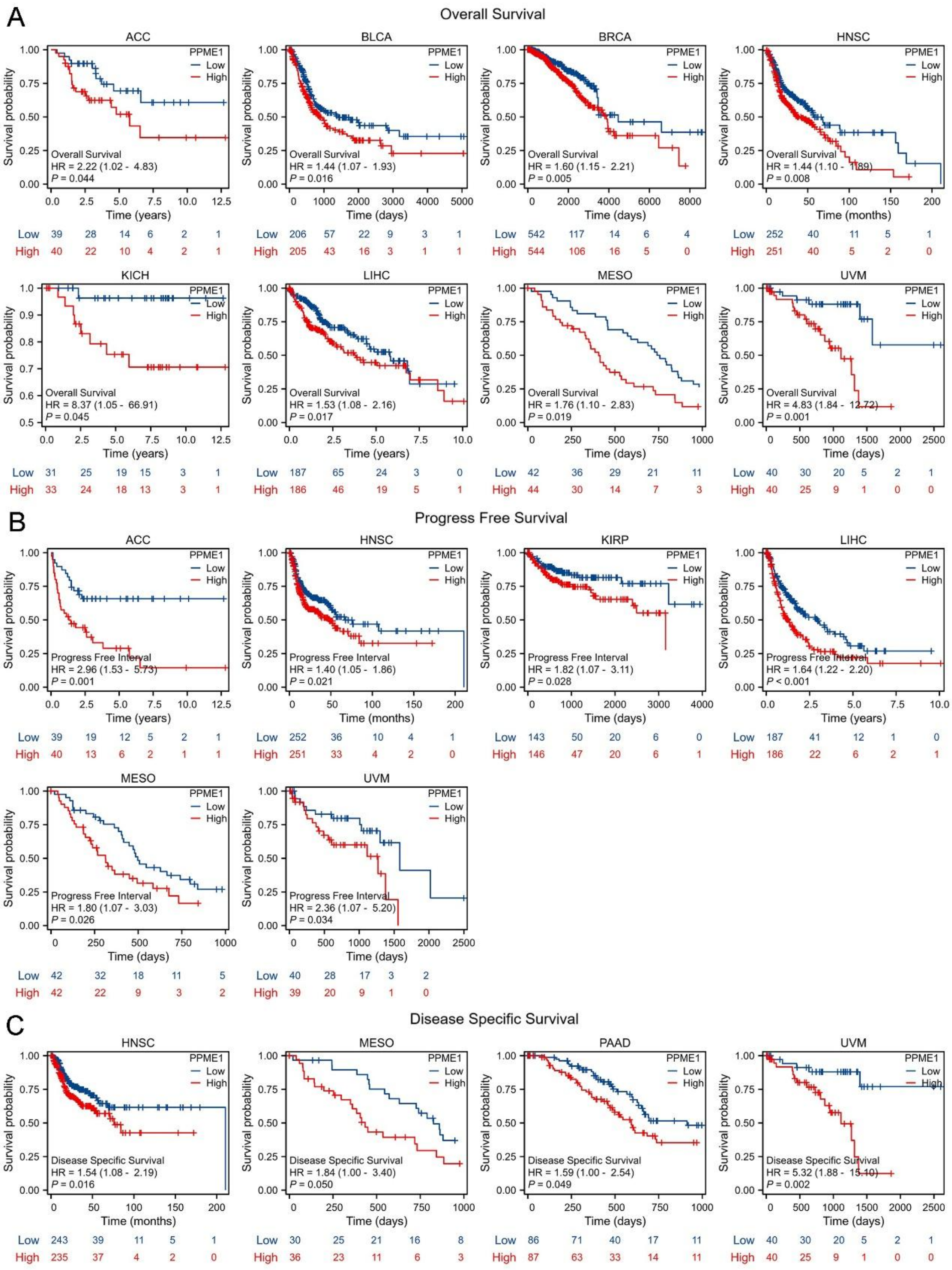

Supplementary Figure S2. Kaplan-Meier (KM) Curves Showing the Detailed Associati on Between TUBA1B Expression and Prognosis in Cancer Patients. **(A)** Overall Survi val (OS) in ACC, BLCA, BRCA, HNSC, KICH, LIHC, MESO, and UVM. **(B)** Progr

ession-free survival (PFS) in ACC, HNSC, KIRP, LIHC, MESO, and UVM. **(C)** Disease-Specific Survival (DSS) in HNSC, MESO, PAAD, and UVM.

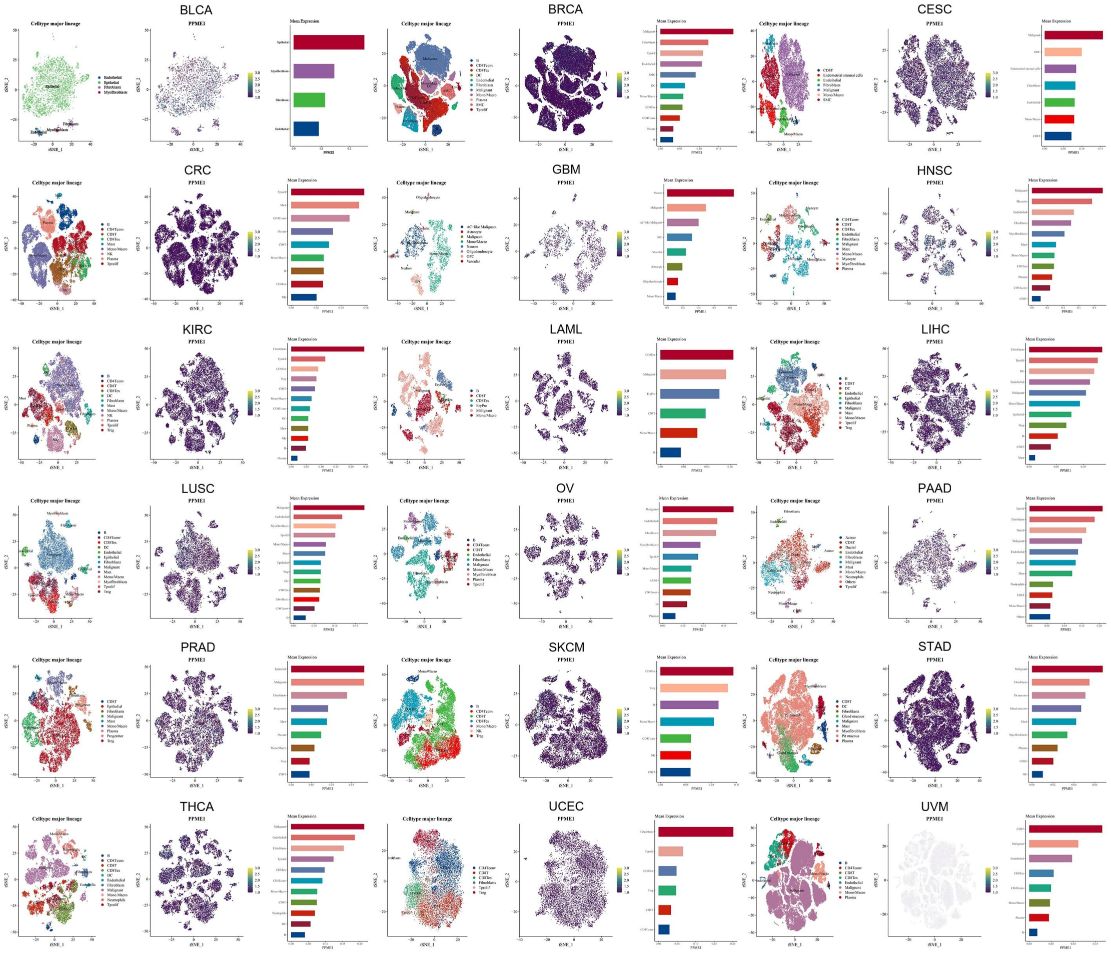

Supplementary Figure S3. Single-cell analysis utilized TISCH database data, featuring cluster and gene expression maps, covering 18 cancer types: BLCA, BRCA, CESC, CRC, GBM, HNSC, KIRC, LAML, LIHC, LUSC, OV, PAAD, PRAD, SKCM, STAD, THCA, UCEC, and UVM.

Supplementary Table S1. PPME1 shRNA sequences and primer sequences.

Name	Targeting sequence(5'→3’)
sh-PPME1-1	GTACAGCTATGGATGCACTTA
sh-PPME1-2	GCTTATCCAATCTCTCTCTCTCTCTCTT A
sh-PPME1-3	GCAGCGATTATTAGT
PPME1-OE-F	TGACATGGAGACCAAGAAAGACC
PPME1-OE-R	ATGCTAGGAAGAGGGGCTCA
PPME1-F	TCAAATGTCTTCCCAGGCTCAG
PPME1-R	CCACCGCTCGCTATGGCTAA
GAPDH-F	ATGGGAAGGTGAAGGTCGG
GAPDH-R	CCTGGAAGATGGTGATGGGATT

Supplementary Table S2. Results of univariate and multivariate Cox analysis of clinic al parameters in ACC **(A)**, BLCA **(B)**, BRCA **(C)**, HNSC **(D)**, KICH **(E)**, LIHC **(F)**, MESO **(G)**, and UVM **(H)**.

A. ACC

Characteristics	Total(N)	Univariate analysis		Multivariate analysis	
		Hazard ratio (95% CI)	P value	Hazard ratio (95% CI)	P value
Pathologic T stage	77				
T1&T2	51	Reference		Reference	
T3&T4	26	10.286 (3.976 - 26.608)	< **0.001**	8.032 (0.972 - 66.369)	0.053
Pathologic N stag e	77				
N0	68	Reference			
N1	9	2.038 (0.769 - 5.400)	0.152		
Clinical M stage	77				
M0	62	Reference		Reference	
M1	15	6.150 (2.710 - 13.959)	< **0.001**	1.133 (0.276 - 4.647)	0.862

Pathologic stage	77				
Stage I&Stage II	46	Reference		Reference	
Stage III&Stage IV	31	6.476 (2.706 - 15.498)	< **0.001**	0.712 (0.072 - 7.055)	0.771
Primary therapy outcome	67				
PD&SD	20	Reference		Reference	
PR&CR	47	0.063 (0.020 - 0.199)	< **0.001**	0.133 (0.035 - 0.504)	**0.003**
Radiation therapy	76				
No	59	Reference			
Yes	17	1.292 (0.512 - 3.262)	0.587		
Age	79				
<= 50	41	Reference			
> 50	38	1.799 (0.846 - 3.824)	0.127		
PPME1	79				
Low	39	Reference		Reference	
High	40	2.222 (1.023 - 4.828)	**0.044**	1.984 (0.618 - 6.372)	0.250

B. BLCA

Characteristics	Total(N)	Univariate analysis		Multivariate analysis	
		Hazard ratio (95% CI)	P value	Hazard ratio (95% CI)	P value
Pathologic T stage	377				
T1&T2	123	Reference		Reference	
T3&T4	254	2.157 (1.485 - 3.132)	< **0.001**	1.541 (0.485 - 4.902)	0.464
Pathologic N stage	367				
N0	238	Reference		Reference	
N1&N2&N3	129	2.250 (1.649 - 3.072)	< **0.001**	1.275 (0.661 - 2.459)	0.469
Pathologic M stag	212				

e					
M0	201	Reference		Reference	
M1	11	3.112 (1.491 - 6.493)	**0.002**	1.216 (0.392 - 3.777)	0.735
Pathologic stage	409				
Stage I&Stage II	133	Reference		Reference	
Stage III&Stage IV	276	2.267 (1.567 - 3.281)	< **0.001**	1.218 (0.342 - 4.333)	0.761
Primary therapy outcome	355				
PD&SD	100	Reference		Reference	
PR&CR	255	0.229 (0.164 - 0.320)	< **0.001**	0.358 (0.193 - 0.667)	**0.001**
Gender	411				
Female	108	Reference			
Male	303	0.868 (0.629 - 1.198)	0.390		
Age	411				
<= 70	231	Reference		Reference	
> 70	180	1.424 (1.064 - 1.906)	**0.018**	1.239 (0.738 - 2.079)	0.417
Radiation therapy	385				
No	364	Reference			
Yes	21	0.967 (0.475 - 1.968)	0.926		
PPME1	411				
Low	206	Reference		Reference	
High	205	1.436 (1.070 - 1.926)	**0.016**	1.896 (1.104 - 3.258)	**0.021**

C. BRCA

Characteristics	Total(N)	Univariate analysis		Multivariate analysis	
		Hazard ratio (95% CI)	P value	Hazard ratio (95% CI)	P value
Pathologic T stage	1,083				
T1&T2	908	Reference		Reference	

T3&T4	175	1.588 (1.096 - 2.301)	**0.014**	0.886 (0.526 - 1.492)	0.649
Pathologic N stage	1,067				
N0	516	Reference		Reference	
N1&N2&N3	551	2.232 (1.563 - 3.189)	**< 0.001**	1.820 (1.169 - 2.832)	**0.008**
Pathologic M stage	925				
M0	905	Reference		Reference	
M1	20	4.266 (2.474 - 7.354)	**< 0.001**	2.243 (1.110 - 4.530)	**0.024**
Pathologic stage	1,062				
Stage I&Stage II	800	Reference		Reference	
Stage III&Stage IV	262	2.367 (1.686 - 3.321)	**< 0.001**	1.645 (0.956 - 2.831)	0.072
Age	1,086				
<= 60	603	Reference		Reference	
> 60	483	2.024 (1.468 - 2.790)	**< 0.001**	2.131 (1.473 - 3.083)	**< 0.001**
PPME1	1,086				
Low	542	Reference		Reference	
High	544	1.597 (1.152 - 2.213)	**0.005**	1.531 (1.062 - 2.208)	**0.023**

D. HNSC

Characteristics	Total(N)	Univariate analysis		Multivariate analysis	
		Hazard ratio (95% CI)	P value	Hazard ratio (95% CI)	P value
Pathologic T stage	447				
T1&T2	179	Reference		Reference	
T3&T4	268	1.934 (1.413 - 2.649)	**< 0.001**	1.930 (1.190 - 3.130)	**0.008**
Pathologic N stage	410				
N0	170	Reference		Reference	

N1&N2&N3	240	1.853 (1.334 - 2.572)	**< 0.001**	1.685 (1.144 - 2.481)	**0.008**
Pathologic stage	435				
Stage I&Stage II	94	Reference		Reference	
Stage III&Stage IV	341	1.839 (1.236 - 2.737)	**0.003**	1.145 (0.517 - 2.536)	0.738
Gender	503				
Female	134	Reference			
Male	369	0.760 (0.571 - 1.012)	0.061		
Age	503				
<= 60	247	Reference			
> 60	256	1.262 (0.964 - 1.653)	0.090		
PPME1	503				
Low	252	Reference		Reference	
High	251	1.441 (1.100 - 1.889)	**0.008**	1.464 (1.062 - 2.020)	**0.020**

E. KICH

Characteristics	Total(N)	Univariate analysis		Multivariate analysis	
		Hazard ratio (95% CI)	P value	Hazard ratio (95% CI)	P value
Pathologic T stage	64				
T1&T2	44	Reference		Reference	
T3&T4	20	10.121 (2.098 - 48.814)	**0.004**	11.844 (2.436 - 57.579)	**0.002**
Gender	64				
Female	26	Reference			
Male	38	1.528 (0.381 - 6.125)	0.550		
Age	64				
<= 50	33	Reference			
> 50	31	3.847 (0.797 - 18.561)	0.093		
PPME1	64				
Low	31	Reference		Reference	

High	33	8.366 (1.046 - 66.911)	**0.045**	10.117 (1.259 - 81.332)	**0.030**

F. LIHC

Characteristics	Total(N)	Univariate analysis		Multivariate analysis	
		Hazard ratio (95% CI)	P value	Hazard ratio (95% CI)	P value
Pathologic T stage	370				
T1&T2	277	Reference		Reference	
T3&T4	93	2.598 (1.826 - 3.697)	< **0.001**	2.500 (1.753 - 3.565)	< **0.001**
Pathologic N stag e	258				
N0	254	Reference			
N1	4	2.029 (0.497 - 8.281)	0.324		
Pathologic M stag e	272				
M0	268	Reference			
M1	4	4.077 (1.281 - 12.973)	**0.017**		
Gender	373				
Female	121	Reference			
Male	252	0.793 (0.557 - 1.130)	0.200		
Age	373				
<= 60	177	Reference			
> 60	196	1.205 (0.850 - 1.708)	0.295		
AFP(ng/ml)	279				
<= 400	215	Reference			
> 400	64	1.075 (0.658 - 1.759)	0.772		
PPME1	373				
Low	187	Reference		Reference	
High	186	1.528 (1.079 - 2.164)	**0.017**	1.425 (1.002 - 2.027)	**0.049**

G. MESO

Characteristics	Total(N)	Univariate analysis		Multivariate analysis	
		Hazard ratio (95% CI)	P value	Hazard ratio (95% CI)	P value
Pathologic T stage	84				
T1&T2	39	Reference			
T3&T4	45	0.978 (0.606 - 1.578)	0.927		
Pathologic N stage	82				
N0&N1	54	Reference			
N2&N3	28	0.884 (0.530 - 1.474)	0.637		
Pathologic M stage	60				
M0	57	Reference			
M1	3	1.856 (0.441 - 7.817)	0.399		
Pathologic stage	86				
Stage I&Stage II	26	Reference			
Stage III&Stage IV	60	0.993 (0.588 - 1.675)	0.978		
Gender	86				
Female	16	Reference			
Male	70	0.888 (0.494 - 1.595)	0.691		
Age	86	1.021 (0.991 - 1.051)	0.166		
Radiation therapy	85				
No	60	Reference			
Yes	25	0.682 (0.403 - 1.155)	0.155		
PPME1	86				
Low	42	Reference		Reference	
High	44	1.763 (1.098 - 2.831)	**0.019**	1.763 (1.098 - 2.831)	**0.019**

H. UVM

Characteristics	Total(N)	Univariate analysis		Multivariate analysis	
		Hazard ratio (95% CI)	P value	Hazard ratio (95% CI)	P value
Clinical T stage	78				
T2&T3	40	Reference			
T4	38	2.416 (0.964 - 6.055)	0.060		
Clinical M stage	80				
M0	73	Reference		Reference	
M1&MX	7	4.004 (1.321 - 12.140)	**0.014**	6.405 (1.980 - 20.718)	**0.002**
Clinical stage	80				
Stage II	36	Reference			
Stage III&Stage IV	44	1.718 (0.704 - 4.193)	0.234		
Gender	80				
Female	35	Reference			
Male	45	1.542 (0.651 - 3.652)	0.325		
Age	80				
<= 60	40	Reference			
> 60	40	2.123 (0.914 - 4.933)	0.080		
BMI	53				
<= 30	39	Reference			
> 30	14	1.920 (0.705 - 5.234)	0.202		
PPME1	80				
Low	40	Reference		Reference	
High	40	4.832 (1.835 - 12.721)	**0.001**	6.024 (2.180 - 16.649)	**< 0.001**

Supplementary Table S3. List of the top 100 genes associated with PPME1.

Supplementary Table S4. GO terms and KEGG pathways enriched in the analysis.
