# Supplementary material for: The value of Protein Phosphatase Methylesterase 1 in diagnosis, prognosis and immunoregulation: from pan-cancer analysis to breast cancer verification
Source: Front Immunol. 2026 Mar 10;17:1770711. doi: 10.3389/fimmu.2026.1770711 (PMC13008989; doi:10.3389/fimmu.2026.1770711)
Supplement: Supplementary file 2 [file DataSheet2.pdf]

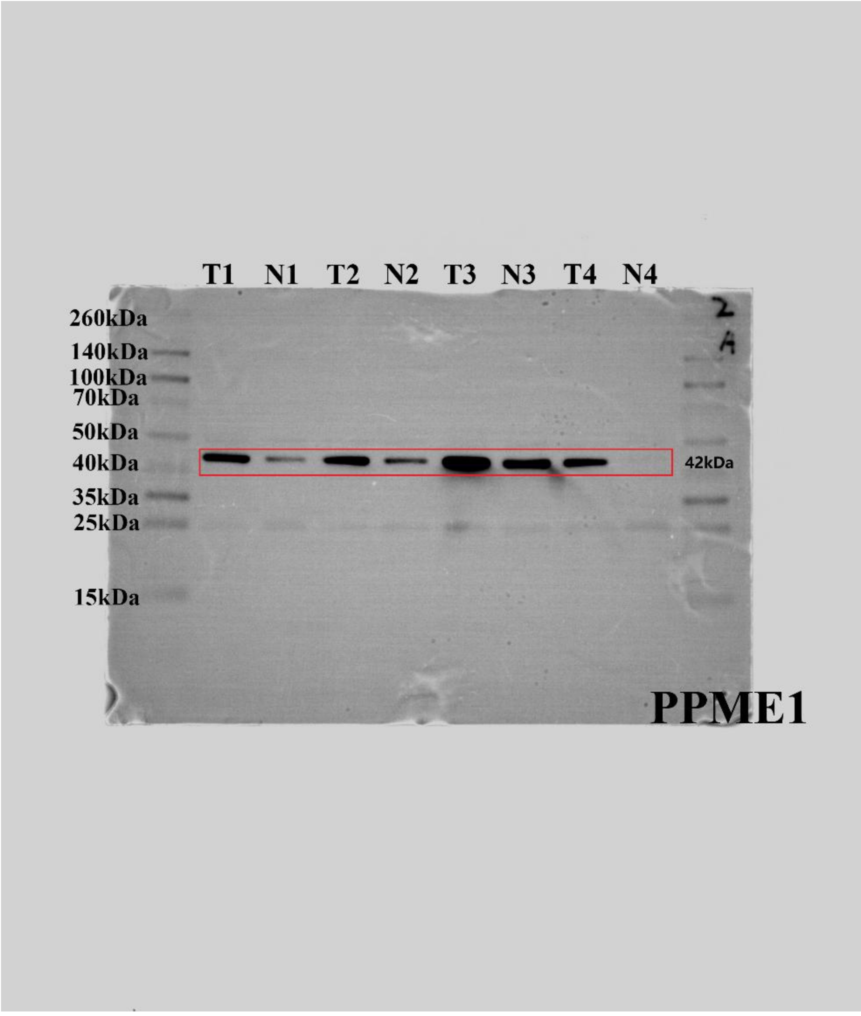

Figure 1. PPME1 blot images of patients 1-4.Lane 1: Cancerous tissue of patient 1 (T1). Lane 2: Paracancerous tissue of patient 1 (N1).Lane 3: T2. Lane 4: N2. Lane 5: T3. Lane 6: N3. Lane 7: T4. Lane 8: N4

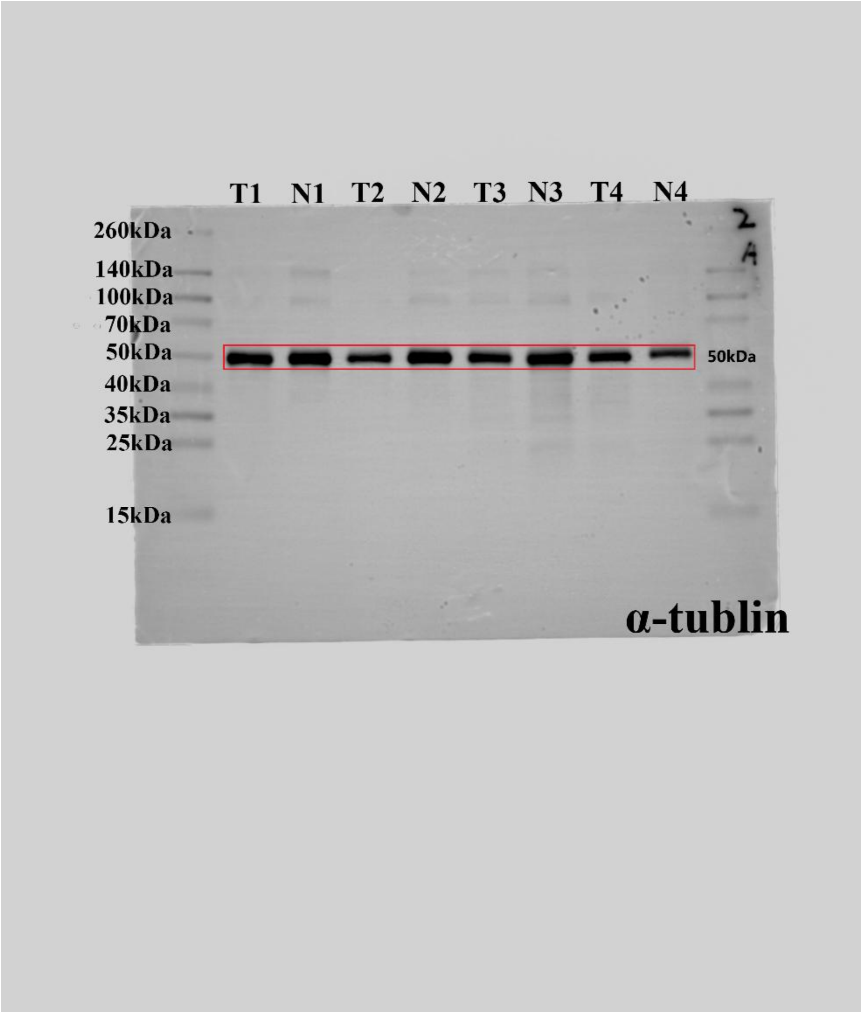

Figure 2. α-tublin blot images of patients 1-4. Lane 1: T1. Lane 2: N1. Lane 3: T2. Lane 4: N2. Lane 5: T3. Lane 6: N3. Lane 7: T4. Lane 8: N4.

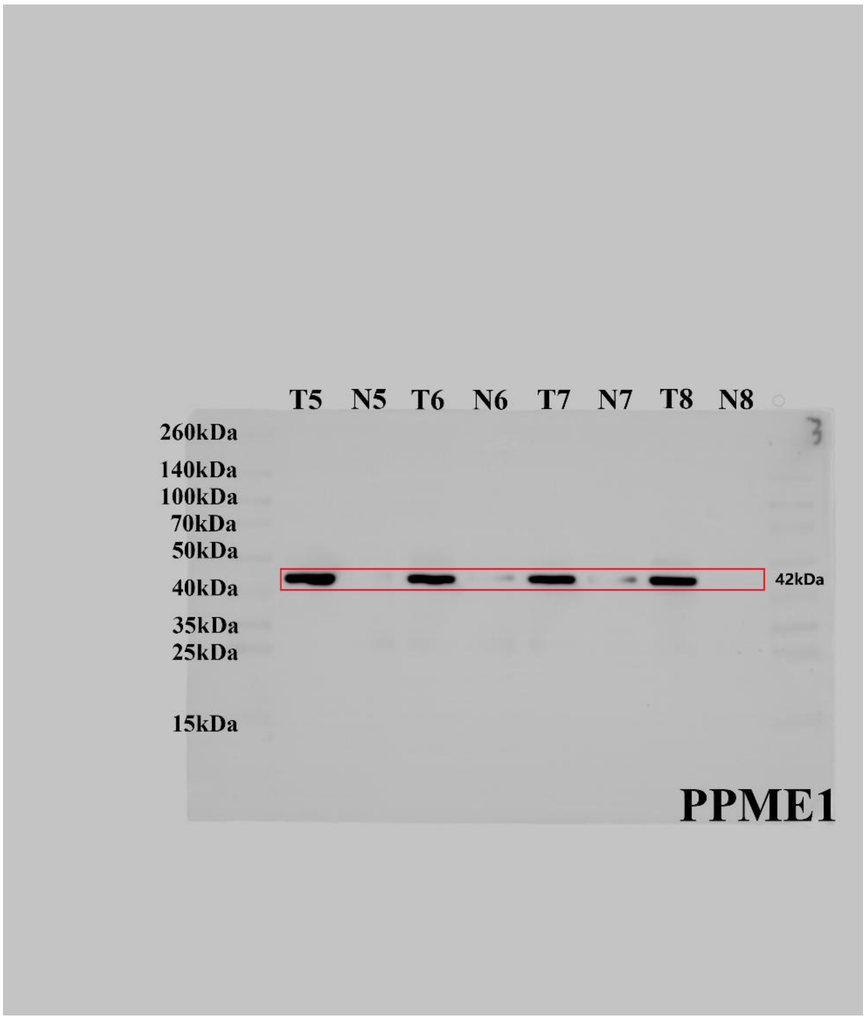

Figure 3: PPME1 blot images of patients 5-8.Lane 1: Cancerous tissue of patient 1 (T5). Lane 2: Paracancerous tissue of patient 1 (N5).Lane 3: T6. Lane 4: N6. Lane 5: T7. Lane 6: N7. Lane 7: T8. Lane 8: N8

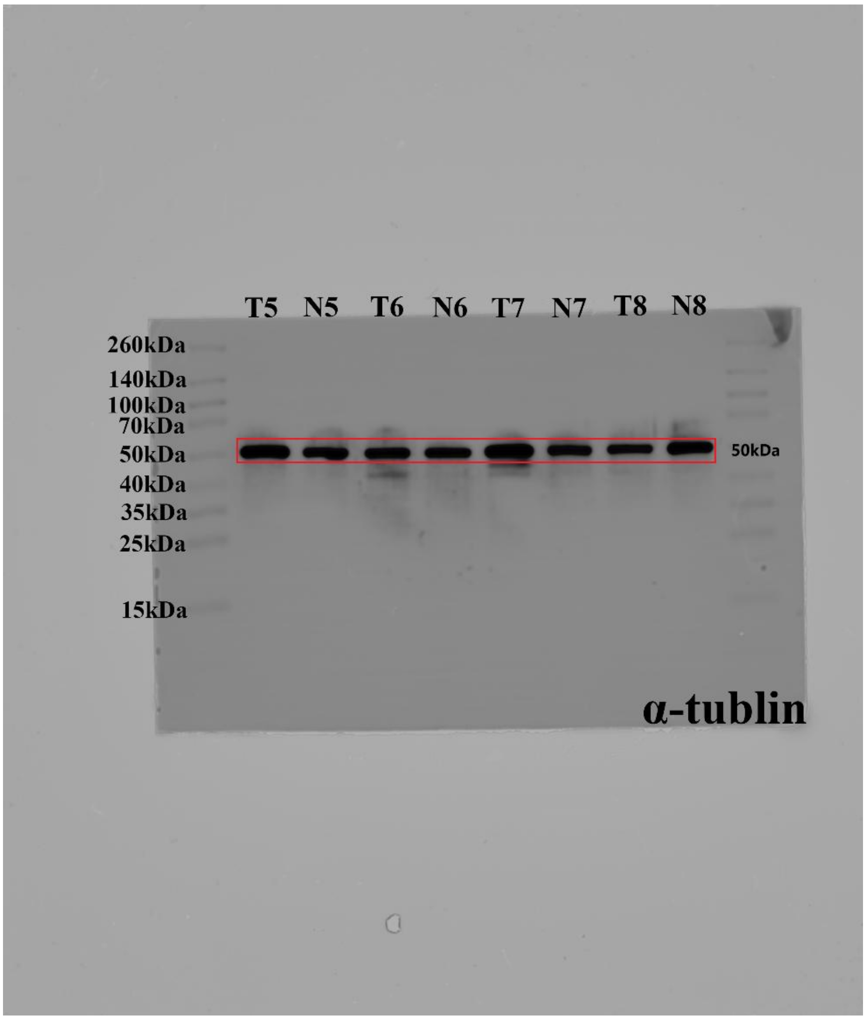

Figure 4. α-tublin blot images of patients 5-8. Lane 1: T5. Lane 2: N5. Lane 3: T6. Lane 4: N6. Lane 5: T7. Lane 6: N7. Lane 7: T8. Lane 8: N8.

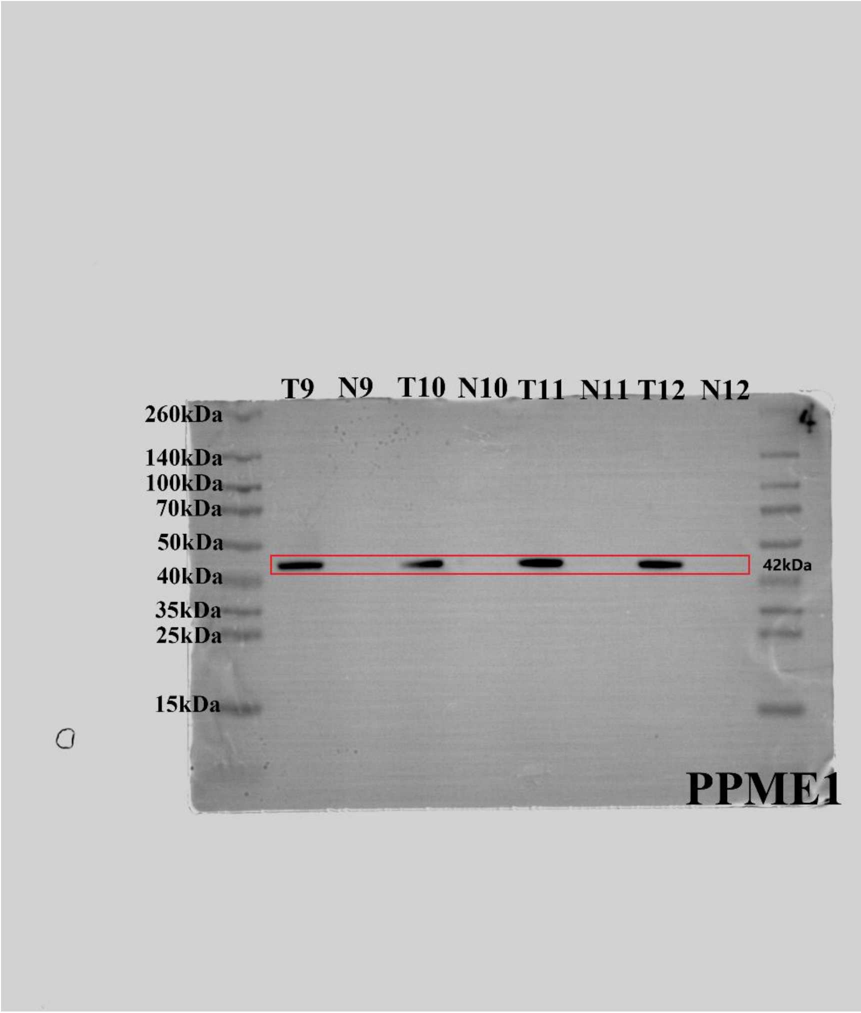

Figure 5: PPME1 blot images of patients 9-12.Lane 1: Cancerous tissue of patient 1 (T9). Lane 2: Paracancerous tissue of patient 1 (N9).Lane 3: T10. Lane 4: N10. Lane 5: T11. Lane 6: N11. Lane 7: T12. Lane 8: N12

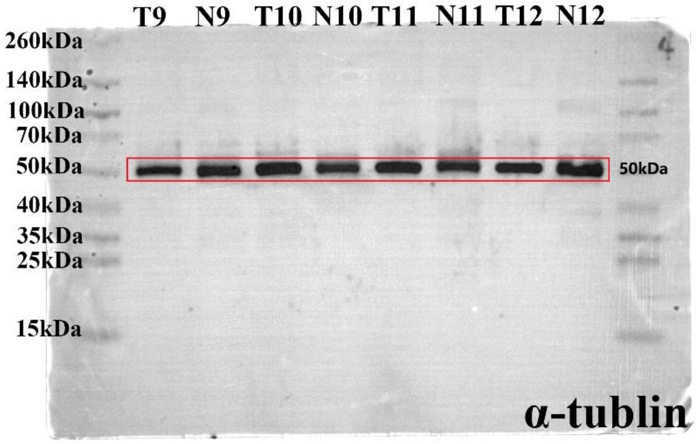

Figure 6: α-tublin blot images of patients 9-12. Lane 1: T9. Lane 2: N9. Lane 3: T10. Lane 4: N10. Lane 5: T11. Lane 6: N11. Lane 7: T12. Lane 8: N12

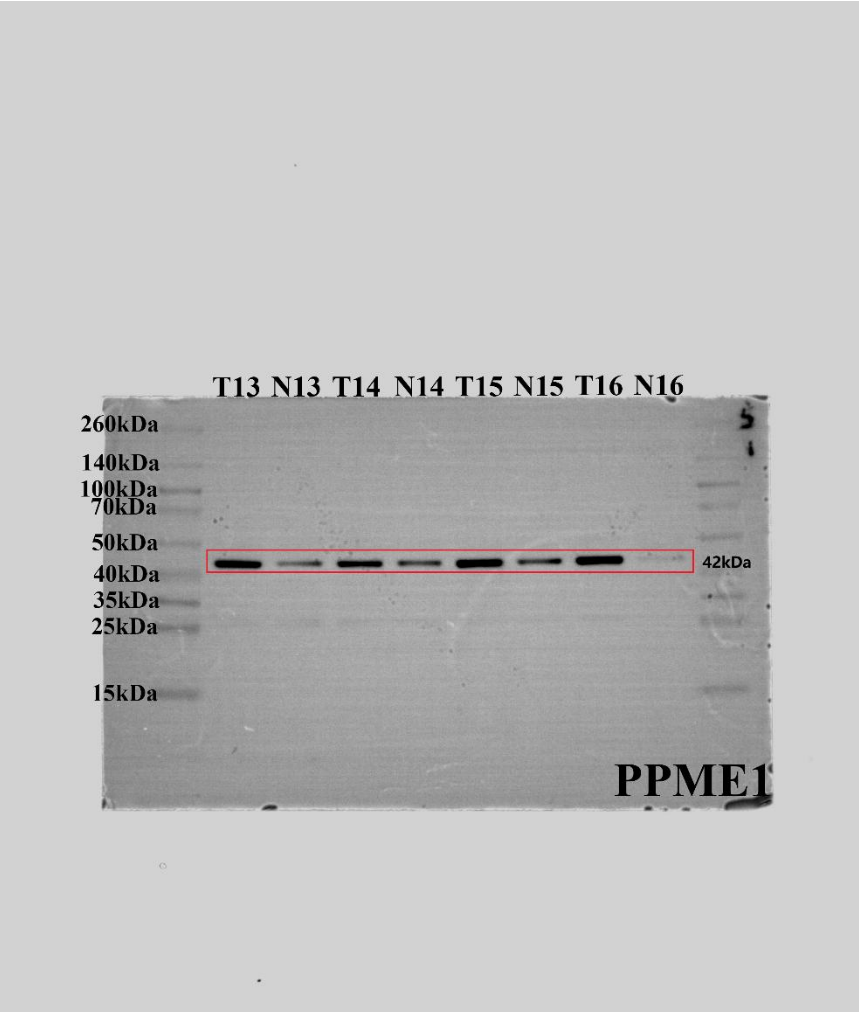

Figure 7: PPME1 blot images of patients 13-16.Lane 1: Cancerous tissue of patient 1 (T13). Lane 2: Paracancerous tissue of patient 1 (N13).Lane 3: T14. Lane 4: N14. Lane 5: T15. Lane 6: N15. Lane 7: T16. Lane 8: N16

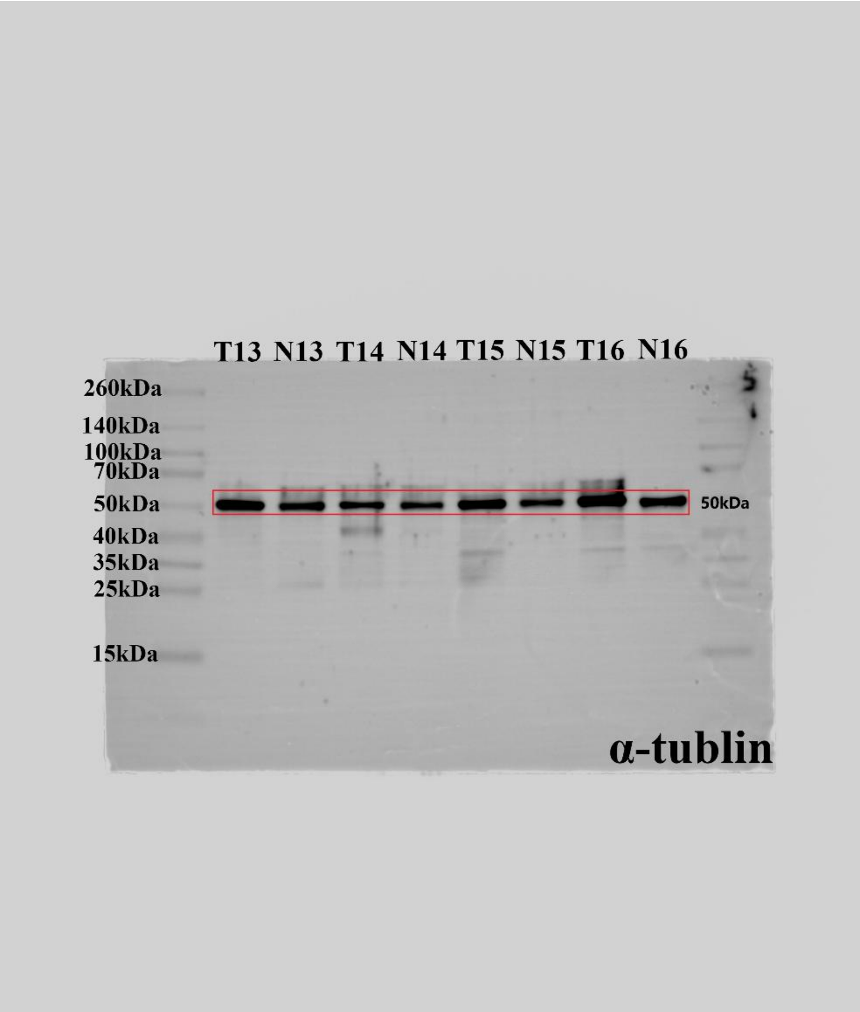

Figure 8： α-tublin blot images of patients 13-16. Lane 1: T13. Lane 2: N13. Lane 3: T14. Lane 4: N14. Lane 5: T15. Lane 6: N15. Lane 7: T16. Lane 8: N16

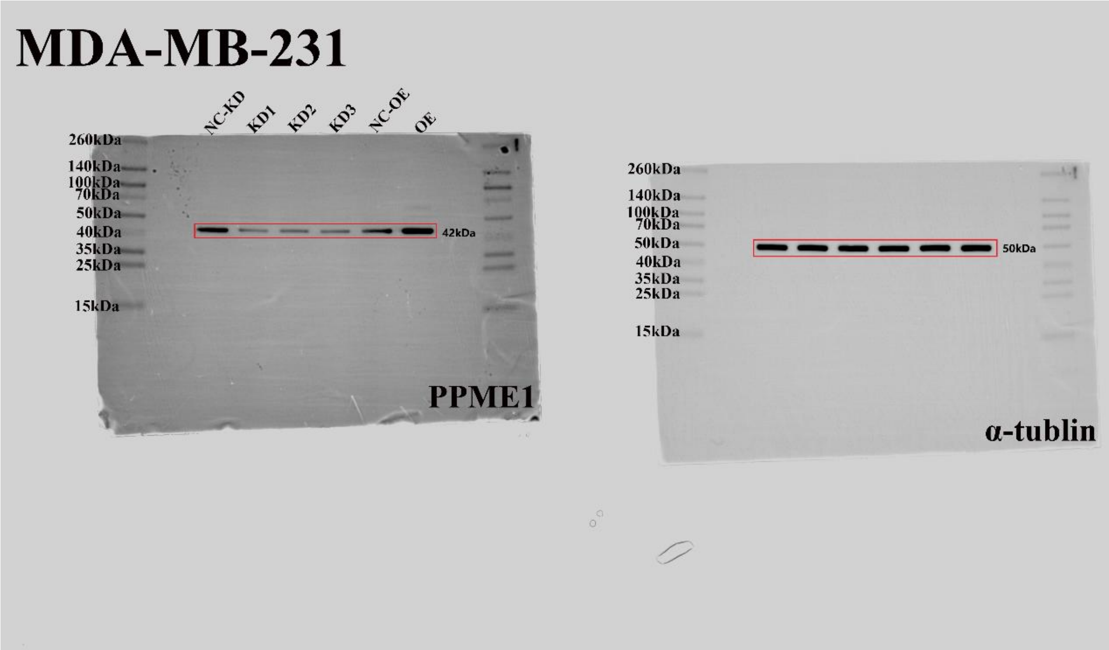

Figure 9： Western blot original images of PPME1 knockdown or overexpression in breast cancer cell MDA-MB-231

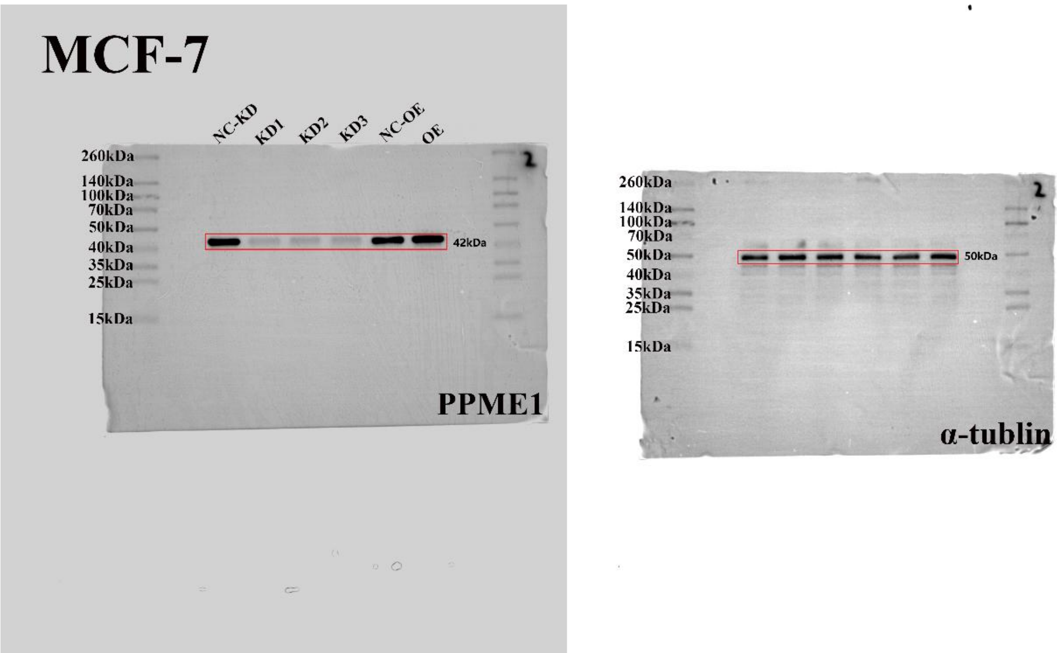

Figure 10： Western blot original images of PPME1 knockdown or overexpression in breast cancer cell MCF-7
