# Supplementary material for: The value of Protein Phosphatase Methylesterase 1 in diagnosis, prognosis and immunoregulation: from pan-cancer analysis to breast cancer verification
Source: Front Immunol. 2026 Mar 10;17:1770711. doi: 10.3389/fimmu.2026.1770711 (PMC13008989; doi:10.3389/fimmu.2026.1770711)
Supplement: Supplementary file 3 [file DataSheet3.pdf]

S-4（2.0版）

新疆医科大学第一附属医院医学伦理委员会审批报告

伦理审批号：230714-07

研究项目名称	基于TIGIT/CD155轴探讨三阴乳腺癌免疫微环境中CD8+T细胞免疫逃逸机制研究		
研究科室	乳腺外科	主要研究者	郭晨明
审查文件	1. 伦理审查申请书<br>2. 项目申请书<br>3. 知情同意书（3.0版）<br>4. 人类遗传资源不涉及出境声明		
伦理审查方式	■会议审查 □快速审查	项目来源	纵向课题
投票结果	同意	主审委员	刘剑
审查说明	伦理委员会对该项目申请等相关材料进行了会议审查，审查投票结果为“同意”，经伦理委员会委员审查认为该研究符合伦理原则，同意开展。		
审查结果及意见	注：1.本研究跟踪审查频率为12个月，请定期向伦理委员会提交跟踪审查报告表。若发生严重不良事件，请及时提交严重不良事件报告。<br>2.本批件有效期为自批准之日起1年。<br>3.研究开始前，请申请人完成医学研究登记备案信息系统登记注册。<br>4.如对研究方案或知情同意书进行修改应及时向伦理委员会报告，所涉及的伦理问题均应提交伦理委员会再次审议。<br><br>**在研究过程中，研究者应遵守国际《赫尔辛基宣言》及我国四部委联合印发的《涉及人的生命科学和医学研究伦理审查办法》与GCP等相关的伦理原则、道德标准及相关的法律、法规、常规、制度等。**<br><br>主任或副主任委员签字：[signature]<br>医学伦理委员会（盖章）<br>日期：2023 年 7 月 9 日		

附件：□NA　　□伦理委员会名单签到及保密协议
