# Supplementary material for: The value of Protein Phosphatase Methylesterase 1 in diagnosis, prognosis and immunoregulation: from pan-cancer analysis to breast cancer verification
Source: Front Immunol. 2026 Mar 10;17:1770711. doi: 10.3389/fimmu.2026.1770711 (PMC13008989; doi:10.3389/fimmu.2026.1770711)
Supplement: Supplementary file 5 [file DataSheet5.pdf]

新疆医科大学第一附属医院动物实验医学伦理委员会审批报告

Approval Letter of Ethics Committee of First Affiliated Hospital of Xinjiang Medical University

研究审批号	A240301-194	审批日期	2024-03-01
研究项目名称	TRIM28 介导微管蛋白 TUBA1B SUMO 化修饰参与三阴性乳腺癌生长转移的作用及机制研究		
项目负责人	郭晨明	中心/科室	消化血管外科中心乳腺外科
项目来源	2024 年国家自然科学基金申报项目		
审查说明	动物实验医学伦理委员会对该研究的相关材料进行快速审查，经审查认为该研究符合伦理原则，**准予申报，如获得立项支持，请再次向伦理委员会递交相关材料进行会议审查。**		
审批意见	在研究过程中，研究者应严格遵循实验动物的福利伦理原则，随时接受新疆医科大学第一附属医院实验动物伦理与使用委员会（IACUC）的监督与检查。<br><br>**主任委员/副主任委员签名：**[illegible]<br><br>**2024 年 3 月 01 日**		
