# Supplementary material for: The value of Protein Phosphatase Methylesterase 1 in diagnosis, prognosis and immunoregulation: from pan-cancer analysis to breast cancer verification
Source: Front Immunol. 2026 Mar 10;17:1770711. doi: 10.3389/fimmu.2026.1770711 (PMC13008989; doi:10.3389/fimmu.2026.1770711)
Supplement: Supplementary file 4 [file DataSheet4.zip › KL43850-1.doc]

AATTCTGGCCGTTTTTGGCTTTTTTGTTAGACGAAGCTTGGGCTGCAGGTCGACTCTAGAGGATCCCGCCACCATGTCGGCCCTCGAAAAGAGCATGCACCTCGGCCGCCTTCCCTCTCGCCCACCTCTACCCGGCAGCGGGGGCAGTCAGAGCGGAGCCAAGATGCGAATGGGCCCTGGAAGAAAGCGGGACTTTTCCCCTGTTCCTTGGAGTCAGTATTTTGAGTCCATGGAAGATGTAGAAGTAGAGAATGAAACTGGCAAGGATACTTTTCGAGTCTACAAGAGTGGTTCAGAGGGTCCAGTCCTGCTCCTTCTGCATGGAGGAGGTCATTCTGCCCTTTCTTGGGCTGTGTTCACGGCAGCGATTATTAGTAGAGTTCAGTGTAGGATTGTAGCTTTGGATCTGCGAAGTCATGGTGAAACAAAGGTCAAGAATCCTGAAGATCTGTCTGCAGAAACAATGGCAAAAGACGTTGGCAATGTGGTTGAAGCCATGTATGGGGACCTTCCTCCTCCAATTATGCTGATTGGACATAGCATGGGTGGTGCTATTGCAGTCCACACAGCATCATCCAACCTGGTACCAAGCCTCTTGGGTCTGTGCATGATTGATGTTGTAGAAGGTACAGCTATGGATGCACTTAATAGCATGCAGAATTTCTTACGGGGTCGTCCTAAAACCTTCAAGTCTCTGGAGAATGCTATTGAATGGAGTGTGAAGAGTGGCCAGATTCGAAATCTGGAGTCTGCCCGTGTCTCAATGGTTGGCCAAGTCAAACAGTGTGAAGGAATTACAAGTCCAGAAGGCTCAAAATCTATAGTGGAAGGAATCATAGAGGAAGAAGAAGAAGATGAGGAAGGAAGTGAGTCTATAAGCAAGAGGAAAAAGGAAGATGACATGGAGACCAAGAAAGACCATCCATACACCTGGAGAATTGAACTGGCAAAAACAGAAAAATACTGGGACGGCTGGTTCCGAGGCTTATCCAATCTCTTTCTTAGTTGTCCCATTCCTAAATTGCTGCTCTTGGCTGGTGTTGATAGATTGGATAAAGATCTGACCATTGGCCAGATGCAAGGGAAGTTCCAGATGCAGGTCCTACCCCAGTGTGGCCATGCAGTCCATGAGGATGCCCCTGACAAGGTAGCTGAAGCTGTTGCCACTTTCCTGATCCGGCACAGGTTTGCAGAACCCATCGGTGGATTCCAGTGTGTGTTTCCTGGCTGTACCGGTATGGACTACAAGGATGACGATGACAAGGATTACAAAGACGACGATGATAAGGACTATAAGGATGATGACGACAAATGAGCTAGCCTGTGGAATGTGTGTCAGTTAGGGTGTGGAAAGTCCCCAGGCTCCCCAGCAGGCAGAAGTATGCAAAGCATGCATCTCAATT
