# Supplementary material for: The value of Protein Phosphatase Methylesterase 1 in diagnosis, prognosis and immunoregulation: from pan-cancer analysis to breast cancer verification
Source: Front Immunol. 2026 Mar 10;17:1770711. doi: 10.3389/fimmu.2026.1770711 (PMC13008989; doi:10.3389/fimmu.2026.1770711)
Supplement: Supplementary file 9 [file Table4.docx]

**Cover Letter**

Dear Editor,

I would like to submit a paper entitled ‘**The value of Protein Phosphatase Methylesterase 1 in diagnosis, prognosis and immunoregulation: from pan-cancer analysis to breast cancer verification**’ to your appreciation and to the editorial analysis of the ***Frontiers in Immunology*.**

Worldwide, cancer is the leading cause of death. Despite the development of multiple antitumor therapies, new cancer treatment strategies require research on specific genes and their associations with different malignancies. The Protein Phosphatase Methylesterase 1 (PPME1) is a methylesterase specific to phosphatase 2A, a tumor suppressor, and plays a key role in tumor development. Herein, to investigate the possibility of PPME1 as a novel immunotherapy strategy for tumor treatment, we did a thorough structure/functional pan-carcinoma investigation of it, which included omics analysis, prognostic analysis, epigenetic analysis, immunoassay analysis, and enrichment analysis. Our study suggests that PPME1 could potentially serve as a diagnostic marker for predicting cancer immunological profiles and survival outcomes and shed light on the expression and role of PPME1 in breast cancer, providing a solid foundation for considering it as a promising therapeutic target for breast cancer patient treatment.

I declare that the paper has not been submitted or is not simultaneously being submitted elsewhere. The full article has been read and approved by all authors. No conflicts of interest exit in the submission of this manuscript. If accepted, it will not be published elsewhere including electronically in the same form, in English or in any other language, without the written consent of the copyright-holder.

Sincerely yours,

Chenming Guo (Professor) and Dilimulati Ismtula (Professor)

The First Affiliated Hospital of Xinjiang Medical University

No. 137 Liyushan South Road

Urumqi, Xinjiang

P.R.China

830054
